# Roles of long non-coding RNAs and emerging RNA-binding proteins in innate antiviral responses

**DOI:** 10.7150/thno.48520

**Published:** 2020-07-23

**Authors:** Yiliang Wang, Yun Wang, Weisheng Luo, Xiaowei Song, Lianzhou Huang, Ji Xiao, Fujun Jin, Zhe Ren, Yifei Wang

**Affiliations:** 1 Guangzhou Jinan Biomedicine Research and Development Center, Institute of Biomedicine, College of Life Science and Technology, Jinan University, Guangzhou 510632, PR China.; 2 Key Laboratory of Virology of Guangzhou, Jinan University, Guangzhou 510632, P.R, China.; 3 Department of Obstetrics and Gynecology, The First Affiliated Hospital of Jinan University, Guangzhou 510632, PR China.

**Keywords:** long non-coding RNAs, RNA-binding proteins, innate antiviral responses, N^6^-methyladenosine, TRIM family

## Abstract

The diseases caused by viruses posed a great challenge to human health, the development of which was driven by the imbalanced host immune response. Host innate immunity is an evolutionary old defense system that is critical for the elimination of the virus. The overactive innate immune response also leads to inflammatory autoimmune diseases, which require precise control of innate antiviral response for maintaining immune homeostasis. Mounting long non-coding RNAs (lncRNAs) transcribed from the mammalian genome are key regulators of innate antiviral response, functions of which greatly depend on their protein interactors, including classical RNA-binding proteins (RBPs) and the unconventional proteins without classical RNA binding domains. In particular, several emerging RBPs, such as m^6^A machinery components, TRIM family members, and even the DNA binding factors recognized traditionally, function in innate antiviral response. In this review, we highlight recent progress in the regulation of type I interferon signaling-based antiviral responses by lncRNAs and emerging RBPs as well as their mechanism of actions. We then posed the future perspective toward the role of lncRNA-RBP interaction networks in innate antiviral response and discussed the promising and challenges of lncRNA-based drug development as well as the technical bottleneck in studying lncRNA-protein interactions. Our review provides a comprehensive understanding of lncRNA and emerging RBPs in the innate antiviral immune response.

## Introduction

As highlighted by the current COVID-19 pandemic, the virus posed a constant threat to global human health, diseases caused by which are closely associated with immune disorders 1. The host immune system includes innate immunity and adaptive immunity, the former of which is the first line of defense against invasive pathogens 2-4. However, the overactive innate immune response would damage the host tissues 2, 5. The relatively long-lasting innate antiviral response must, therefore, be precisely tuned to maintain immune homeostasis. Although several regulators of the innate antiviral immunity have been identified, the mechanisms of fine-tuning of the innate antiviral response remain obscure. The mammalian genome can be transcribed into vast long non-coding RNAs (lncRNAs), which are important modulators in a variety of physiological and pathological processes 6, 7. Mounting lncRNAs are gradually identified as key regulators in innate antiviral response and virus infection 7-13. Indeed, virus infection greatly changes the expression profile of the host cell genome, especially the non-coding transcripts 14. lncRNAs were defined as non-coding RNAs with at least 200 nucleotides in length 15. Based on the location relative to protein-coding genes (P-CGs), the conventional lncRNAs include five classes: (i) long intergenic transcripts are separated by transcriptional units from P-CGs; (ii) intronic lncRNAs locate within the intron of P-CGs; (iii) bidirectional lncRNAs are transcribed in opposite directions with the promoter of P-CGs; (iv) antisense lncRNAs are transcribed across the exons of a P-CGs from the opposite direction; and (v) pseudogene-type lncRNAs are transcribed from a gene without the ability to produce proteins 7, 15. The unconventional lncRNAs are representative by those transcripts whose stability maintained by a mature 3′ end of a U-A-U triple-helix structure generated by RNase P cleavage, by capping by snoRNA-protein complexes or by forming covalently closed circular structures 6. The achievement of lncRNAs functions greatly depends on their protein interactors including typical RNA-binding proteins (RBPs) and unconventional RBPs 6, 7, 16-20. Importantly, there is novel RBPs gradually found to be as crucial regulators in innate antiviral response 18, 19, 21. These RBPs mainly include N^6^-methyladenosine (m^6^A) machinery components (e.g. heterogeneous nuclear ribonucleoprotein A2/B1, hnRNPA2B1), tripartite motif (TRIM) family members (e.g. TRIM25), and even those DNA binding factors recognized traditionally (e.g. cGAS). However, the lncRNA interactors of most RBPs associated with virus infection remains unknown. Significantly, the mutant of the gene-encode lncRNAs (e.g. SNORA31) or the deficiency of the RNA lariat metabolism-associated gene (e.g. DBR1) can result in virus infection-associated encephalitis as demonstrated by clinical samples 22, 23. Collectively, the lncRNA-RBPs interaction networks would be a brave new world of the regulation of innate antiviral immunity. Herein, in this review, we portray the importance of lncRNAs and emerging RBPs in innate antiviral response as well as their mechanism of actions. Also, the corresponding promising and challenges for the development of lncRNA-based drugs would be discussed. Our review would be beneficial for understanding the function of lncRNAs and RBPs in virus pathogenesis and provide novel insight into the future research of RBPs in innate antiviral response.

## Innate antiviral response

Innate immunity is the first and most rapid line of defense against the invasion of microbial pathogens 2, 5, 24. Host cells mount innate immune response once recognized the conserved virus components termed pathogen-associated molecular patterns (PAMPs) and damage-associated molecular patterns (DAMPs) via pattern recognition receptors (PRRs) 24, 25. PAMPs are usually the conserved molecular components essential for pathogen survival such as nucleic acids, lipopolysaccharide (LPS), lipoproteins, and bacterial flagellin 2, 24, 25. In the cases of the virus, the well-recognized PAMPs are viral genomes and viral nucleic acids generated during the virus replication in the host 5. By contrast, PRRs present either on the cellular surface and within specific cellular compartments of the cytosol as well as the nucleus at steady state 24. PRRs mainly included Toll-like receptors (TLRs), retinoic acid-like receptors (RLRs), cytosolic DNA sensors, the nucleotide-binding and oligomerization, leucine-rich proteins (NLRs), and absent in melanoma 2(AIM2)-like receptors (ALRs) 24, 25. Upon recognizing viral PAMPs, PRRs would be activated and then initiate the downstream innate signaling for the production of type I interferons(IFNs) and /or multiple cytokines and chemokines, causing the synthesis of various antiviral proteins 5, 24. The secreted cytokines and chemokines also recruit immune cells to the sites with virus infection to initiate the adaptive immune response to control virus infection 5. We did not discuss the initiation of adaptive immunity as it is beyond this review.

### DNA/RNA sensors-mediated expression of type I IFNs

**Figure 1:**
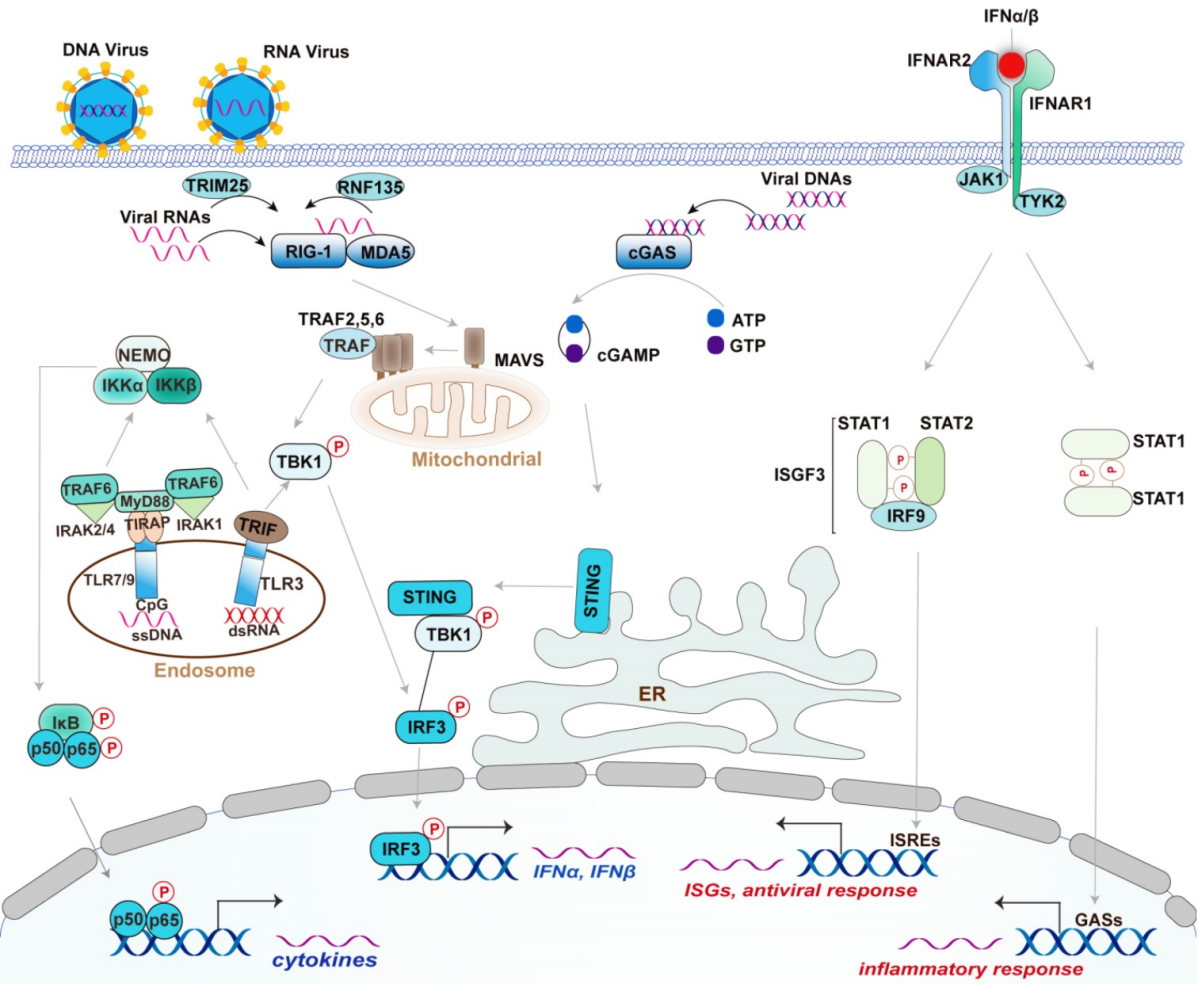
** Canonical Type I IFN signaling activated by DNA virus and RNA virus.** Upon recognized viral RNA, RIG-I is activated by TRIM25- and RNF135-mediated K63 ubiquitination and translocated to mitochondrial at which activates MAVS. Activated MAVS undergoes self-polymerized then recruits a group of ubiquitin ligase TRAF2, TRAF5, and TRAF6 to activate downstream kinases TBK1 and IKK. TBK1 activation induced the expression of type I IFN by activating transcriptional factor IRF3, whereas IKK complex activation induces the expression of proinflammatory cytokines by activating NF-κB. TLR3 recognizes dsRNA and triggers IRF3 and NF-κB signaling through the adaptor protein TRIF; Upon DNA virus infection, cGAS recognizes viral DNA then synthesizes cGAMP from ATP and GTP. cGAMP induces the activation and trafficking of STING to the sites at which recruits TBK1 and activates it to induce the production of type I IFNs by activating IRF3. TLR9 mainly recognizes unmethylated CpG DNA and activates NF-κB through the adaptors MyD88 and TIRAP. Activated TLR9 also initiates an alternative MyD88-dependent signaling pathway that activates the transcription factor IRF7 to induce the expression of type I IFNs in DCs (not depicted). The secreted IFNα and IFNβ bind to the interferon-α receptor IFNAR that composed of IFNAR1 and IFNAR2 subunits. The adaptor kinase JAK1 and TYK2 are activated by this binding and then recruit STAT complex as indicated. The ISGF3 complex is composed of STAT1, STAT2, and IRF9, which binds to the ISRE elements to activate ISGs. By contrast, the STAT1 homodimers bind to GASs elements to induce the production of inflammatory mediators. Type I IFNs also activates STAT3 homodimers, which represent a repressor of inflammatory pathways (not depicted).

The RNA sensors are mainly the RLR family members, including Retinoic acid-inducible gene I (RIG-I), Melanoma differentiation-associated gene 5 (MDA5), and Laboratory of genetics and physiology 2 (LGP2) 24, 26. The RNA characters recognized by them are different. Specifically, RIG-I recognizes the triphosphate and diphosphate at the end of a double-stranded RNA (dsRNA) stem 24, 27, while MDA5 recognizes the internal duplex structure of dsRNA 24, 28. By contrast, LGP2 lacks the caspase activation and recruitment domains (CARDs) required for activating downstream signaling but shares homology at its DExD/H RNA helicase domain and C-terminal domain (CTD) with RIG-I and MDA5 24, 29. LGP2 appears to make the viral RNA more accessible to RIG-I and MDA5 24, 29. These RLRs are generally crucial for host defense against RNA virus; however, RIG-I also functions in defense against some DNA virus with the assistance of RNA polymerase III detecting cytosolic DNA 30. Once recognizing the ligands, RIG-I is modified with K63-linked ubiquitin by tripartite motif (TRIM)-containing 25 (TRIM-25) and RIPLET (also termed RNF135) 31-33 (**Figure 1**). With an aid from protein chaperone 14-3-3ε, the modified RIG-I is translocated to the limited membranes of mitochondria, peroxisomes, and the mitochondria-associated membranes (MAMs) 34-37, at which interacts and activates the mitochondrial-resident adaptor MAVS via CARD domain within RIG-I. Activated MAVS undergoes CARD-dependent self-polymerization and then recruits a series of ubiquitin ligases including TRAF2, -5, and -6, which are required for activating downstream kinases, such as TBK1 and the IKK complex 38, 39. These kinases regulate various transcription factors NF-κB, IRF3, and IRF7, culminating in the expression of IFN, ISGs, and proinflammatory factors 24. TLR3, an endosomal TLR, recognizes viral double-strand RNA (dsRNA) from some viral genomes and replication intermediates, which are uncommon in the mammalian 40. Unlike RLRs, TLR3 responds to dsRNA and triggers downstream signaling through the adaptor protein TRIF, while it can similarly activate IRF3 to produce type I IFN, and NF-κB to produce proinflammatory cytokines 40. TLR3 also plays redundant protective immunity against DNA virus HSV-1 via recognizing the intermediate dsRNA produced by HSV-1 during its life cycle 41, 42.

The DNA sensors in mammalian cells mainly include cGAS, ALRs such as AIM2 and IFI16, and TLR9. Upon recognizing DNA, cGAS utilizes ATP and GTP to synthesize the cyclic di-GMP/AMP (cGAMP), a cyclic dinucleotide harboring a high affinity to the adaptor STING (stimulator of IFN genes, also known as MITA, TMEM173, MPYS, and ERIS) 43-45. STING is predominantly localized on the endoplasmic reticulum at the steady-state but undergoes trafficking to poorly defined vesicles or puncta via the Golgi apparatus upon when activated by the binding of cGAMP 43, 46 (**Figure 1**). Following the STING movement, TBK1 and IRF3 activation are initiated, contributing to the production of type I IFNs 43 (**Figure 1**). The helicase DDX41 was also reported to sense intracellular DNA in a STING-dependent manner 47. However, the authors did not investigate the effect of Ddx41 on viral replication using Ddx41 knockout mice to elucidate the essential role of Ddx41 in DNA-mediated innate antiviral response. hnRNPA2B1 is an emerging nuclear-resident DNA sensor 48, which would be introduced in detail in the section of RBPs. By contrast, TLR9 mainly recognizes unmethylated CpG DNA motifs 49. In plasmacytoid DCs (pDCs), TLR9 initiates a MyD88-dependent signaling pathway that activates the transcription factor IRF7 to trigger the production of IFNs 50.

By contrast, AIM2 mainly promotes the inflammasome formation following the intracellular DNA recognition 51-53. Inflammasomes are multiprotein complexes initiating the innate immune response mainly characterized by the secretion of proinflammatory cytokines (IL-1β, IL-18) and pyroptosis, a rapid form of cell death causing further inflammation 54. Given these cytokines were not the leading factors in innate antiviral response, the detailed information regarding inflammasome is not discussed. IFI16 functions in STING-dependent IFN production in response to intracellular DNA 24, 55. IFI16 was distributed in both nucleus and cytosol depending on cell type. Briefly, detection of DNA virus, including herpes simplex virus-1(HSV-1) and KSHV, by IFI16 occurs within the nucleus, whereas the activation of STING by IFI16 occurs in the cytosol 24. However, IFI16 is not an essential factor for the IFN response to DNA virus infection 55.

### The canonical type I IFN signaling pathway

Type I IFNs, especially IFN-α and IFN-β, initiates the inflammatory response and transcription of antiviral genes such as IFN-stimulated genes (ISGs) 4. In brief, both IFN-α and IFN-β bind the IFN-α receptor (IFNAR), a heterodimeric transmembrane receptor composed by IFNAR1 and IFNAR2 subunits, and then activates the receptor-associated protein tyrosine kinases Janus kinase 1(JAK1) and tyrosine kinase (TYK2), culminating in the phosphorylation of signal transducer and activator of transcription 1 (STAT1) and STAT2 4, 56, 57. The phosphorylated-STAT1 and STAT2 then dimerize and enter into the nucleus at which form IFN-stimulated gene factor 3 (ISGF3) complex by the assemble with IFN-regulatory factor 9 (IRF9) 4, 57. Consequently, ISGF3 binds to the IFN-stimulated response elements (ISREs), thereby activating the transcription of IFN-stimulated genes (ISGs), such as IFN-induced GTP-binding protein and 2ʹ-5ʹ-oligoadenylate synthase (OAS) 4, 57. ISG-encoded proteins showed a great activity of restraining pathogens by the degradation of viral nucleic acids, the inhibition of viral transcription, translation, and replication, and the reprogrammed cellular metabolism 58, 59. Of note, activation of IFNAR by type I IFNs also leads to the formation and nuclear translocation of STAT1 homodimers that subsequently bind to the gamma-activated sequence (GAS) to induce pro-inflammatory genes 4. Collectively, the activation of the JAK-STAT pathway by type I IFNs is essential for the interferon-based establishment of a cellular antiviral state. Of note, cellular IFNAR signaling is augmented or restrained by various feedback mechanisms during the course of an immune response, which have been extensively reviewed 60, 61 and thereby are not discussed here.

### Type III IFN signaling pathways

Type III IFNs are the recently found members of the IFN cytokine family and engage a receptor complex formed by the IL-28 R α/IFN-λ R1 ligand-binding subunit and the IL-10R beta accessory chain to activate innate antiviral responses 62-64. The type III IFN family consists of four proteins, IL-29/IFN-λ1, IL-28A/ IFN-λ2, IL-28B/IFN-λ3, and IFN-λ4 62-64. Similar with type I IFNs, type III IFNs activate JAK1 and TYK2, leading to the phosphorylation and activation of STAT1 and STAT2 62, 63, 65. Phosphorylated STAT1 and STAT2 associated with IRF9 to form the ISGF3 complex, which subsequently translocate to the nucleus and initiate the expression of ISGs. In addition, IFN-λ proteins can also induce JAK2 phosphorylation and activate other STAT family proteins, as well as MAPK signaling pathways 65. However, MAPK signaling activated by type III IFNs is not the main contributor combating virus infection.

## Roles of lncRNAs in innate antiviral response

**Figure 2:**
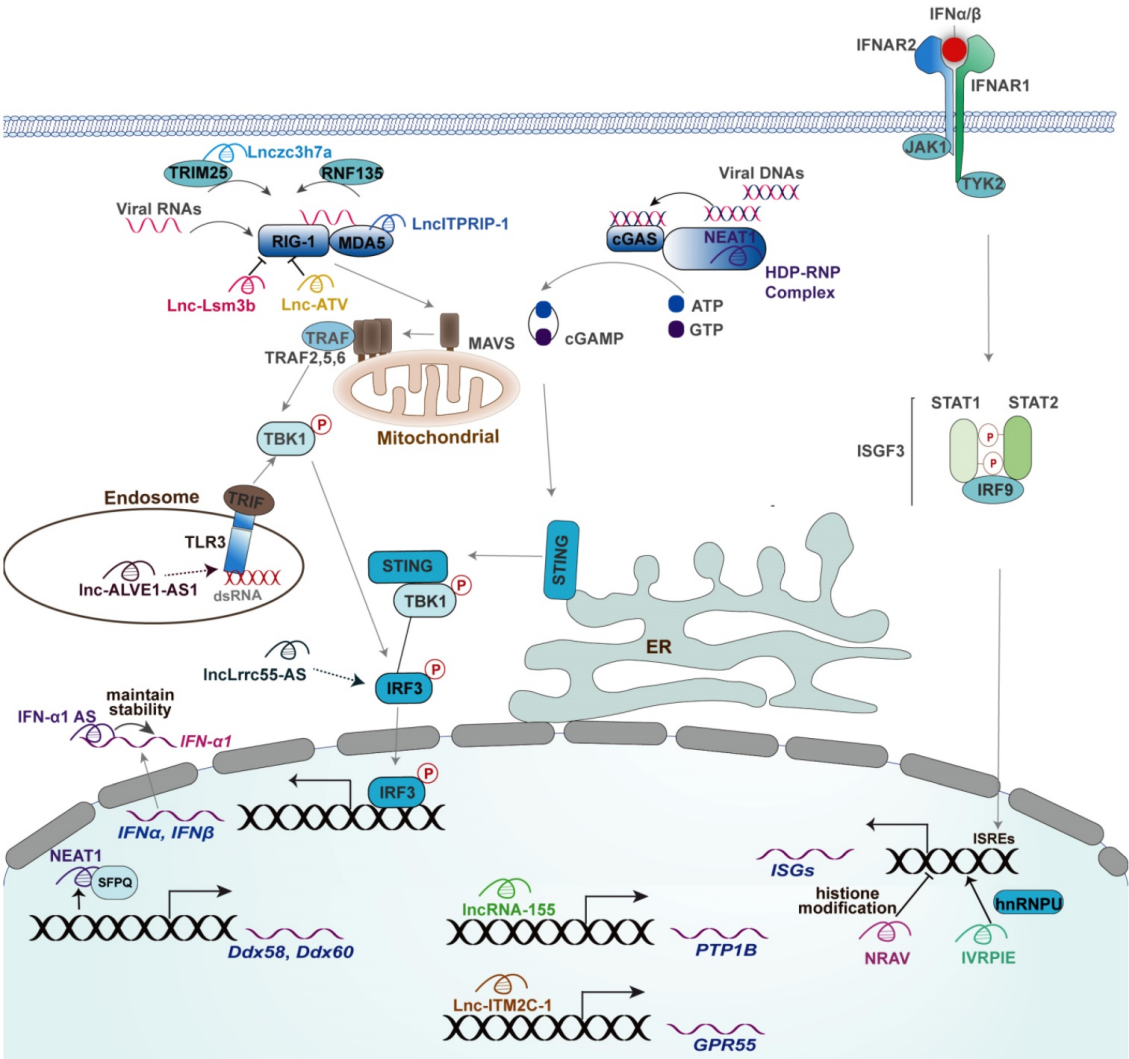
** The mechanisms of actions of lncRNAs in regulating innate antiviral response**. Mouse-derived lncRNA Lnc-Lsm3b inhibits the production of type I IFNs through binding RIG-I to restrict RIG-I conformational shift. lncATV inhibits the expression of type I IFNs through binding RIG-I to restrict RIG-I-mediated innate immunity. Lnczc3h7a promotes a TRIM25-mediated RIG-I antiviral innate immune response. NEAT1 promotes RIG-I and DDX60 expression and facilitates the DNA-dependent activation of the cGAS-STING-IRF3 pathway to upregulate the expression of IFN-β. ITPRIP-1 positively regulates IFN signaling pathway through targeting MDA5. Lnc-ALVE1-AS1 induces an antiviral response by activating the TLR3 signaling. lncLrrc55-AS Promotes I-IFNs signaling by strengthening IRF3 phosphorylation. NRAV negatively regulates the expression of IFITM3 and MxA by affecting histone modification of these genes. IVRPIE promotes the expression of IFN-β and ISGs by modifying their promoter activity through an interaction with hnRNPU. Lnc-ITM2C-1 negatively regulates the expression of ISGs by stimulating expression of GPR55. LncRNA-155 Inhibits the expression of PTP1B and thereby activates TYK2-JAK2 signaling to facilitate the expression of ISGs. The functional lncRNAs with unknown or uncertain mechanisms in innate antiviral responses, including lncRNA-CMPK2, EGOT, and #32, were not depicted.

The transcriptional regulation of cytokine genes in response to pathogen infection lies at the central of immune response research. Numerous lncRNAs are gradually recognized as key factors for virus-host interaction primarily via the antiviral response-dependent and antiviral response-independent manner. The former, as the focus of this review, would be discussed in detail (**Table 1 and Figure 2**), whilst those lncRNAs regulating innate immunity outside the context of virus infection were not enrolled in this review. However, there were currently no studies uncovering the role of lncRNAs in initiating the expression of type III IFNs. Indeed, type I IFNs have a nearly universal antiviral role as compared to type III IFN 66, 67.

### Roles of lncRNAs in modulating the level of type I IFNs

The regulatory roles of lncRNAs in the expression of type I IFNs by the virus are discussed according to the molecular order of PRRs-triggered signaling involved in lncRNAs. Immune recognition of viral components by PRRs is the first step initiating the expression of type I IFNs, at which several lncRNAs act crucial roles; thus, we first discussed the effect of lncRNAs on PRRs, including DNA and RNA sensors. RIG-I is the main RNA sensor in mammalian cells, the release of CARDs within which mediates the downstream signaling for activation of type I IFNs expression 24, 27. A recent study identified a RIG-I-associated host lncRNA term Lnc-Lsm3b in mouse macrophages 8. Specifically, Lnc-lsm3b induced by virus infection directly binds to mice RIG-I within its CTD domain and then restricts its CARDs release and prevents downstream signaling, thereby terminating type I IFNs production 8. However, there was no report of lncRNAs located at the transcript region of* Lsm3b* in the human genome 8, it would be significant for exploring human endogenous lncRNAs like mouse-derived Lnc-Lsm3b that can be recognized by RIG-I. Interestingly, another study reported a human RIG-I-associated lncRNA termed lncATV capable of inhibiting RIG-I-mediated type I IFNs initiation 68. However, whether the binding of lncATV to RIG-I restricts the conformational change of RIG-I, inhibits the ability of binding viral dsRNA by RIG-I, or both of which, needs to be further determined 68. TRIM25-mediated K63-linked ubiquitination of RIG-I within its two CARDs is essential for the formation of RIG-I oligomers that interacts with MAVS to elicit the production of type I IFNs against RNA virus 31. Such a modification can be greatly enhanced by an intronic lncRNA named Lnczc3h7a 20. In addition to the conformational change and post-translation modification, the expression of RIG-I also can be modulated by several lncRNAs, such as NEAT1 69. In detail, NEAT1 relocates SFPQ to paraspeckles and thereby removes the transcriptional inhibitory effects by SFPQ on the transcription of RIG-I 69. Indeed, the RNA sensor MDA5 also can be modulated by a lncRNA named lncITPRIP-1. LncITPRIP-1 enhances the type I IFN signaling response to viral infection by boosting the oligomerization and activation of MDA5 70. Besides, lncITPRIP-1 functions as a cofactor for the binding of MDA5 to HCV RNA 70.

Given most of the RNA sensors-associated factors showed a high affinity with RNA, it is not unreasonable that there were relatively few lncRNAs functioning in DNA sensor-mediated initiation of type I IFNs upon virus infection. In detail, lncRNA NEAT1 is required for the activation of the cGAS-STING-TBK1-IRF3 pathway in response to foreign DNA 72. Such achievement largely depended on the interaction of HEXIM1-DNA-PK-paraspeckle components-ribonucleoprotein complex (HDP-RNP) with cGAS and its partner PQBP1 72 (**Figure 2**). The foreign DNA remodeled this complex, leading to the release of paraspeckle proteins, recruitment of STING, and activation of IRF3 72. However, the interaction of cGAS with NEAT1 remains unknown as this study did not explore cGAS-NEAT1 interaction using RIP or RNA pull-down assay 72. Indeed, a recent study uncovered the RNA-binding activity of cGAS in the exhaustion of dormant hematopoietic stem cells but not in the context of virus infection, while cGAS is a typical DNA-binding protein 81. Indeed, NEAT1 also induces the expression of Interleukin(IL)-8 through relocating SFPQ from the promoter region of IL-8 to paraspeckle upon immune stimuli, including HSV-1 infection 82, whereas the effect of which on virus replication remains uncertain due to the lack of corresponding experiments. TLR3 signaling-induced antiviral response is also associated with a lncRNA named lnc-ALVE1-AS1, an endogenous retrovirus-derived lncRNA 73. However, the detailed mechanism of action of lnc-ALVE1-AS1 remains unknown as this study only tested the effect of lnc-ALVE1-AS1 on the expression of TLR3 73.

In addition to PRRs, the downstream signaling initiated by PRRs also can be regulated by lncRNA. For instance, an interferon-inducible lncRNA named lncLrrc55-AS can strengthen IRF3 activation facilitating antiviral type I IFNs to combat both DNA virus and RNA virus, including SeV, HSV-1, VSV, and IAV 74. Their mechanism study revealed that the binding of lncLrrc55-AS to phosphatase methylesterase 1 (PME-1) enhances the interaction of PME-1 with the phosphatase PP2A and thereby facilitates PME-1-mediated demethylation and inactivation of PP2A to restore the inhibition effect of PP2A on IRF3 phosphorylation 74. Indeed, the detailed mechanisms of action of some lncRNAs that implicated in viral infection-associated diseases in innate antiviral response remain obscure. A recent influential study revealed the mutation of a small nucleolar RNA-encoding gene* SNORA31* in five patients with HSV-1 encephalitis 23. The neurons with such a mutant are susceptible to numerous neurotropic viruses, such as VZV, MeV, poliovirus, VSV, and EMCV, which can be rendered by exogenous IFN-β 23, suggesting a potential role of SNORA31 in the innate antiviral response mediated by IFN-β. However, the detailed mechanism of action of SNORA31 needs to be further explored.

Indeed, most lncRNAs originate from within a 2 kb region surrounding the transcription start sites (TSSs) of P-CGs or to map to enhancer regions 6, 83. This supports that lncRNAs may play major roles in epigenetic regulation, including transcriptional regulation in cis- or trans- and in the organization of nuclear domains 6. However, another crucial role of lncRNAs was also highlighted in the field of post-transcriptional gene regulation, for which lncRNAs leave the site of transcription and operate in trans 6, 84, 85. Trans-acting lncRNAs may function by modulating the abundance or activity of RNAs to which they directly bind in a stoichiometric manner 6. For instance, the natural antisense transcript (NAT)-mRNA regulatory network promotes target mRNA stability by acting in a competing endogenous RNA (ceRNA) manner to form a transient duplex between their common microRNA response element and the corresponding microRNA, thereby inhibiting miRNA-induced mRNA decay 6, 84, 85. Additionally, the NAT can stabilize target mRNA by pairing to a single-stranded loop region formed by the mRNA in the cytoplasm. Such RNA:RNA duplex formation could initiate conformational changes in the sense RNA structure that enhance the accessibility of a stabilizing RBP, thereby modulating RNA stability. Such a regulatory role of lncRNAs was also observed in the post-transcriptional regulation of type I IFN expression. For instance, IFN-alpha1 AS RNA maintains IFN-alpha1 mRNA stability by preventing the microRNA (miRNA)-induced destabilization of IFN-alpha1 mRNA due to the masking of the miR-1270 binding site 66.

### Functions of lncRNAs in type I IFNs signaling

LncRNAs regulate expression of interferon-stimulated genes (ISGs) mainly via targeting upstream transcription factors and epigenetic modification. Specifically, lncRNA #32 is required for the binding of activating transcription factor (ATF2) to the consensus sequence within IRF7 and facilitates the expression of IRF7, which induces the expression of numerous ISGs, including IP-10, RSAD2, CCL5, CXCL11, and OASL 75. However, there was no investigation into the restoration of the lncRNA #32-mediated induction of these ISGs by IRF7 knockout 75. lncRNA-155 inhibits the expression of PTP1B, a factor promoting the dephosphorylation of TYK2 and JAK2, leading to an augmentation of TYK2-JAK2 signaling to facilitate the expression of ISGs including Mx1, IFIT1, ISG15, IFI27, OAS3, and IFITM3^76^. However, this study failed to explore whether PTP1B knockout abolished the inhibition effect of lncRNA-155 on the expression of these ISGs 76. Moreover, lncRNA-155 overexpression also resulted in a significant upregulation of IFN-β 76; thus, the possibility that the increased expression of ISGs resulting from the enhanced IFN-β expression cannot be excluded. Similarly, lnc-ITM2C-1 stimulates the expression of its neighboring gene GPR55, downregulating the expression of ISGs in turn 79, which needs to be more rigorously confirmed.

Several lncRNAs, such as NRAV and IVRPIE, regulate the expression of ISGs in a mechanism of epigenetic modification. In detail, NRAV enhances the modification of histone 3 lysine 27 trimethylation (H3K27me3, a suppression mark) and reduces the modification of histone 3 lysine 4 trimethylation (H3K4me3, an active mark) of the TSSs in multiple critical ISGs, such as IFITM3 and MxA 10. Consequently, NRAV negatively regulates the initial transcription of ISGs 10. Similarly, another lncRNA named IVRPIE positively regulates the expression of various ISGs, including IRF1, IFIT1, IFIT3, Mx1, ISG15, and IFI44L, by increasing H3K4me3 and impairing H3K27me3 in TSSs of these genes 77. However, IVRPIE also affects the expression of IFN-β 77; therefore, the effect of lncRNA IVRPIE on IFN-β may affect the expression of ISGs. In addition to host-encoded lncRNAs, many viruses themselves generate lncRNAs implicated in their life cycle 7. For instance, an HSV-1-encoded lncRNA termed LAT can downregulate the components of the JAK-STAT pathway during the latency infection 86, 87. Together, there are currently no lncRNAs with direct interaction with the crucial factors of JAK-STAT signaling in response to viral infection. Of note, a lncRNA termed lnc-DC controls the differentiation of human dendritic cells by binding STAT3 88. The lnc-DC-STAT3 interaction promotes STAT3 phosphorylation at amino acid position tyrosine-705 by preventing the binding and dephosphorylation of SHP1 88. Indeed, mounting lncRNAs are involved in the regulation of innate immunity, whereas their functions in viral infection and innate antiviral response remain unknown. For example, heterogeneous nuclear ribonucleoprotein (hnRNP) L and hnRNP A/B are associated with the induction of immunity genes TNF-α and CCL5 via an interaction with lncRNA THRIL and lincRNA-Cox2, respectively 89, 90.

## Roles of emerging RBPs in innate antiviral immunity

Given lncRNAs represent pivotal regulators and RLRs recognize viral RNA in innate antiviral immune response, it is not unexpected that RBPs play key roles in innate antiviral responses (**Table 2**). In addition to the RNA sensors (above), the associators of RLRs and TLR3 also harbor an activity of binding RNA, such as PACT, STAU1, and PUM1, most of which participate in the regulation of innate antiviral response by modulating corresponding RLRs (**Table 2**). ZAP, also known as PARP13, is an ISG and RBP that selectively binds to CG-dinucleotide-enriched RNA and recruits multiple RNA processing machines to degrade viral RNAs 91-94 and to promote translational repression 95. Such a mechanism is significant for the elimination of the CG-rich virus. Of note, the long isoform of ZAP, termed ZAP-L, which contains an additional C-terminal catalytically inactive poly ADP-ribose polymerase (PARP) domain, functions as an interferon-resolution factor 93. The ability to discriminate viral RNAs from cellular RNAs of RLRs has been identified, whereas whether these RLRs can bind to “self” cellular RNAs, such as lncRNAs, remains largely unknown. It has been revealed that an inducible lncRNA lnc-Lsm3b by RIG-I restricts innate immune response upon RNA virus 8. The downstream adaptor MAVS of RIG-I can be degraded by poly(C)-binding protein 1(PCBP1) and PCBP2 via recruiting the E3 ubiquitin ligase AIP4 96, 97. PCBP2 also interacts with the nucleotide-binding oligomerization domain (NOD)-like receptor X1 (NLRX1) to mediate the NLRX1-induced degradation of MAVS 98. Given that RBP-lncRNA interactions are closely associated with protein function, it would be significant for exploring the host RNAs self-recognized by RNA sensors in the future. Of note, the experimental validation of lncRNA-protein interactions remains time-consuming and expensive, which is a major technical bottleneck in the field of lncRNA protein interaction. However, there are several emerging databases predicting the lncRNA-protein interactions 99-101, which provide a time-saving solution for validating lncRNA-protein interaction. The combination of experimental validation and database prediction would be the heading direction or promising techniques in the future.

Previous studies focused on RBPs harboring classical RNA-binding domains (RBDs), such as the RNA recognition motif (RRM), the cold shock domain (CSD), hnRNP K homology (KH) domain, or DEAD-box helicase domain 19. Of note, it was gradually recognized that numerous proteins lacking conventional RBDs even the DNA binding proteins are identified as the factors harboring an activity of binding RNA 102, which are also implicated in innate antiviral response. Recent proteome-wide studies have uncovered hundreds of additional proteins binding RNA through unconventional RBDs 19. Moreover, a lncRNA termed GAS5 can bind the DNA-binding domain of the glucocorticoid receptor to prevent the receptor from binding to its DNA response element 103, 104, implying the importance of unconventional RBPs in binding RNA. These unconventional RBDs include intrinsically disordered regions, protein-protein interaction platforms, and enzymatic cores 19. The emerging RBPs involvement in innate antiviral response would be discussed in detail as follows.

### Roles of the m^6^A machinery components in innate antiviral response

**Figure 3:**
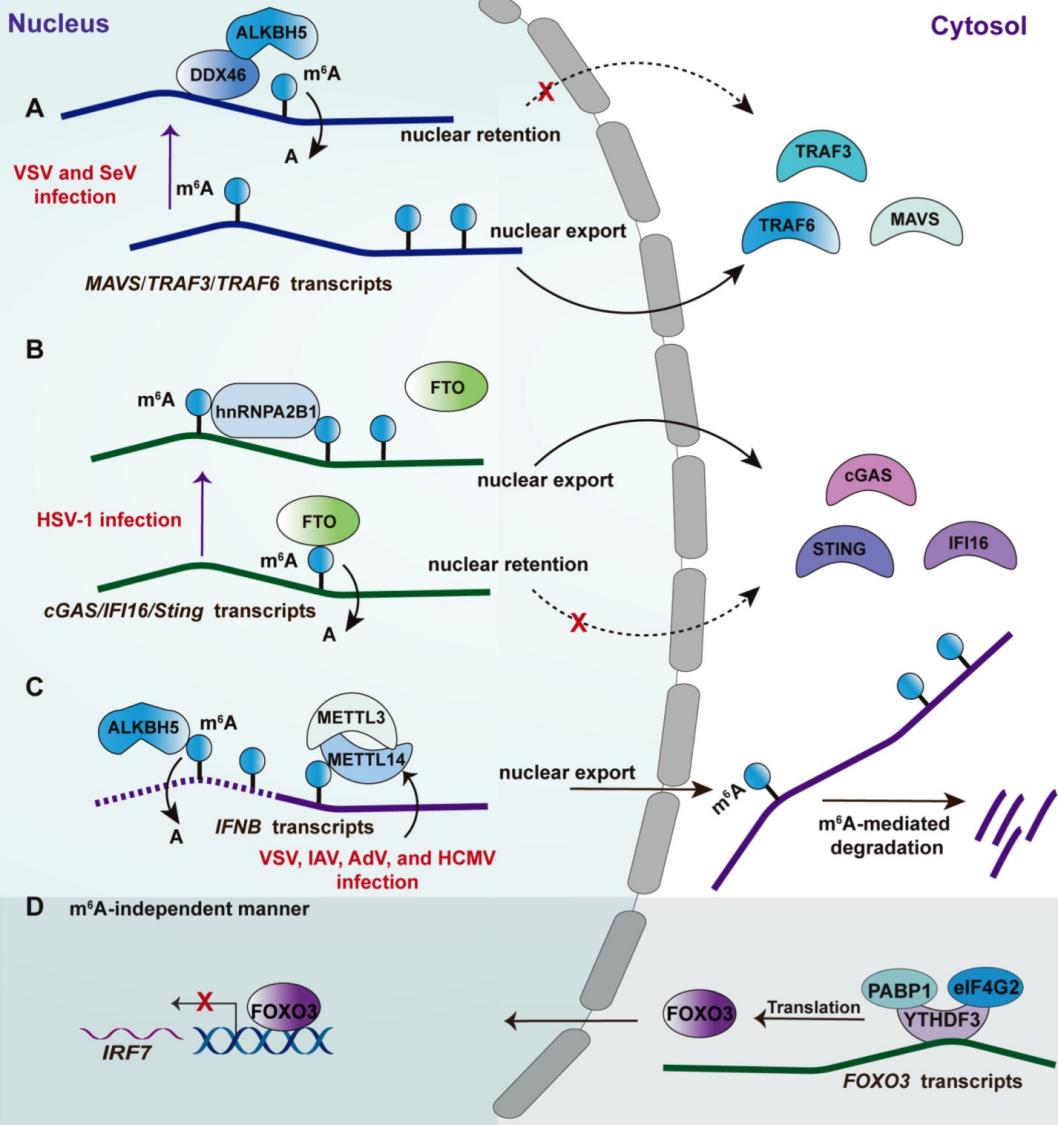
** Effects of m^6^A machinery-associated RBPs on the innate antiviral response.** Upon RNA virus infection, such as VSV and SeV, the RNA helicase DDX46 recruits m^6^A eraser ALKBH5 to remove the m^6^A within *MAVS*, *TRAF3,* and* TRAF6* transcripts, leading to nuclear retention of these transcripts and thereby attenuating type I IFN response. Upon DNA virus infection, such as HSV-1, hnRNPA2B1 limits FTO access to *CGAS*, *STING* and *IFI16* transcripts reducing m^6^A within these antiviral transcripts, leading to their nuclear retention; hnRNPA2B1 also recognizes viral DNA then homodimerizes and undergoes demethylation at Arg226 by JMJD6 to translocate into the cytosol, activating TB1-IRF3 signaling (not depicted). In the context of numerous virus infections, including AdV, HCMV, IAV, and VSV, depletion of the m^6^A writers METTL3-METTL14 heterodimer leads to a reduced level of m^6^A modification of INFB1, counteracting the m^6^A -mediated degradation of IFNB transcripts (dotted line of *IFNB* transcript). Consistently, ALKBH5 can erase the m^6^A preventing the degradation of IFNB transcripts (active line of *IFNB* transcript). Under basal conditions, the m^6^A reader YTHDF3 cooperates with two cofactors, PABP1 and eIF4G2, to promote FOXO3 translation by binding to the translation initiation region of FOXO3 transcripts. Consequently, the FOXO3-IRF7 gene regulatory circuit restrains the type I IFN response and ISG expression. The mechanism was suggested to be m^6^A-independent.

RNA modifications are post-transcriptional regulation by changing the chemical composition of RNAs, including non-coding RNAs and coding RNAs 105. Therefore, most components involvement RNA modification shows a high affinity with RNA by recognizing the corresponding motif. m^6^A is the most prevalent internally modified manner of several identified distinct modifications, the first report regarding which on cellular was in the 1970s 106, 107. The dynamic regulations of m^6^A modification are mainly mediated by dedicated methyltransferase (known as “writer”) and demethylases (known as “eraser”) 105, 108, 109. m^6^A modification influences gene expression post-transcriptionally through altering RNA structure and specific recognition by m^6^A-binding proteins, also known as “readers”^108^. The detailed molecular mechanism of m^6^A had been comprehensively discussed in a previous review 105. Recently, m^6^A modifications have been found to play crucial roles in innate antiviral response as revealed by independent groups. Some m^6^A machinery components also regulate innate antiviral response in an m^6^A-independent manner (**Figure 3**). However, there were some controversial results from these studies linking m^6^A and innate antiviral response. In detail, the Cao group showed that m^6^A promotes innate antiviral immune response (**Figure 3A and 3B**). Specifically, an earlier study from the Cao group reported that DEAD-box helicase 46(DDX46) bound numerous antiviral transcripts, including *Mavs*, *Traf3*, and *Traf6*, via their conserved CCGGUU element 110 (**Figure 3A**). Upon virus infection, DDX46 recruited the 'eraser' ALKBH5 to demethylate these antiviral transcripts 110. The removal of m^6^A led to nuclear retention of these transcripts, leading to a reduction of their protein levels and thereby inhibiting the production of type I IFNs. More recently, the Cao group identified a traditional RBP hnRNPA2B1 as nuclear DNA sensor 48. Upon sensing viral DNA, hnRNPA2B1 homodimerizes and is then demethylated at Arg226 by the arginine demethylase JMJD6 (**Figure 3B**). Such modification leads to the cytoplasm translocation of hnRNPA2B1 to activate the TBK1-IRF3 pathway. Additionally, hnRNPA2B1 facilitates the nucleocytoplasmic trafficking of *IFI16*, *CGAS,* and *STING* mRNAs by enhancing the m^6^A modification of them 48.

Controversially, both the Mohr and Noam group reported that m^6^A weakens the type I IFNs signaling 111, 112 (**Figure 3C**). The transcripts of *IFNB* and *IFNA* are m^6^A-modified and are stabilized following the depletion of the m^6^A writers METTL3 or METTL14. Consistently, depletion of m^6^A “eraser” ALKBH5 reduced the levels of IFNB 112 and led to an increase in viral propagation 111. Moreover, viral replication in a cell with METTL3 or METTL14 deficiency was inhibited in an IFN signaling-dependent manner 111, 112. Of note, another study from the Cao group indicated that m^6^A reader YTHDF3 suppressed ISGs expression, whereas METTL3-mediated m^6^A modification was not involved in such a process 113. However, the possibility of m^6^A mediated by other m^6^A erasers cannot be excluded in this study 113. The mechanism study revealed that YTHDF3 promotes FOXO3 translation by binding to the translation initiation region within FOXO3 transcripts with the cooperation of co-factors PABP1 and eIF4G2 113 (**Figure 3D**). Consequently, the FOXO3 inhibits the transcription of the *IRF7* gene to limit the transcription of type I IFNs as a regulatory circuit 114. The different results from these research groups may be attributed to several reasons: 1) The m^6^A-modified transcript mediated by these m^6^A machinery components are not only limited to antiviral transcripts but also include these transcripts that translated into the cell metabolism-associated factors, which also can be modulated by viral infection affecting viral replication. For example, a recent study by the Cao group found that ALKBH5 demethylates the α-ketoglutarate dehydrogenase (OGDH) transcript, which reduces its stability and protein expression, decreasing the production of metabolite itaconate that is required for viral replication 115; 2) The m^6^A machinery enzymes that are deficient in their studies are different, which would introduce the other effects but not alone the m^6^A mediated by these factors, such as YTHDF3 mentioned above; 3) It has been uncovered that m^6^A modification was found in the genome or transcripts of a broad spectrum of the virus, including positive-sense and negative-sense RNA virus, DNA virus, and retroviruses. Therefore, the consequent effect of m^6^A on virus replication should be jointly attributed to the regulation of antiviral immune response and the direct effect on viral RNA 107. Collectively, the effect of m^6^A modification on these identified transcripts via point mutations can provide more rigorous evidence dissecting the contribution of m^6^A to the corresponding phenotype. The viral RNA modification is also a key manner regulating innate immune. The recruitment of FTSJ3, a 2′-O-methyltransferase, to HIV RNA through TRBP enhances the 2′-O-methylation of the viral genome 116. The viral RNA with such modification cannot be recognized by the RNA sensor MDA5 116, leading to an impaired innate antiviral response.

### Functions of the RNA-binding domain of TRIM-family members in innate antiviral immunity

TRIM proteins constitute a large, diverse, and ancient protein family which play central roles in innate antiviral response that were mostly known and studied based on their ubiquitination activity as E3 ligases 136. However, the TRIM family members have recurrently been cataloged as the novel RBPs due to the RNA binding activity of their NHL or PRY/SPRY domains 20, 21, 137-139. These domains are also crucial for their critical roles in innate antiviral response 17, which would be discussed in detail as follows. Their ability to act both post-transcriptionally and post-translationally is ideally suited to these steps during which cellular states must undergo rapid and dramatic changes, such as the immune response to virus infection.

**Figure 4:**
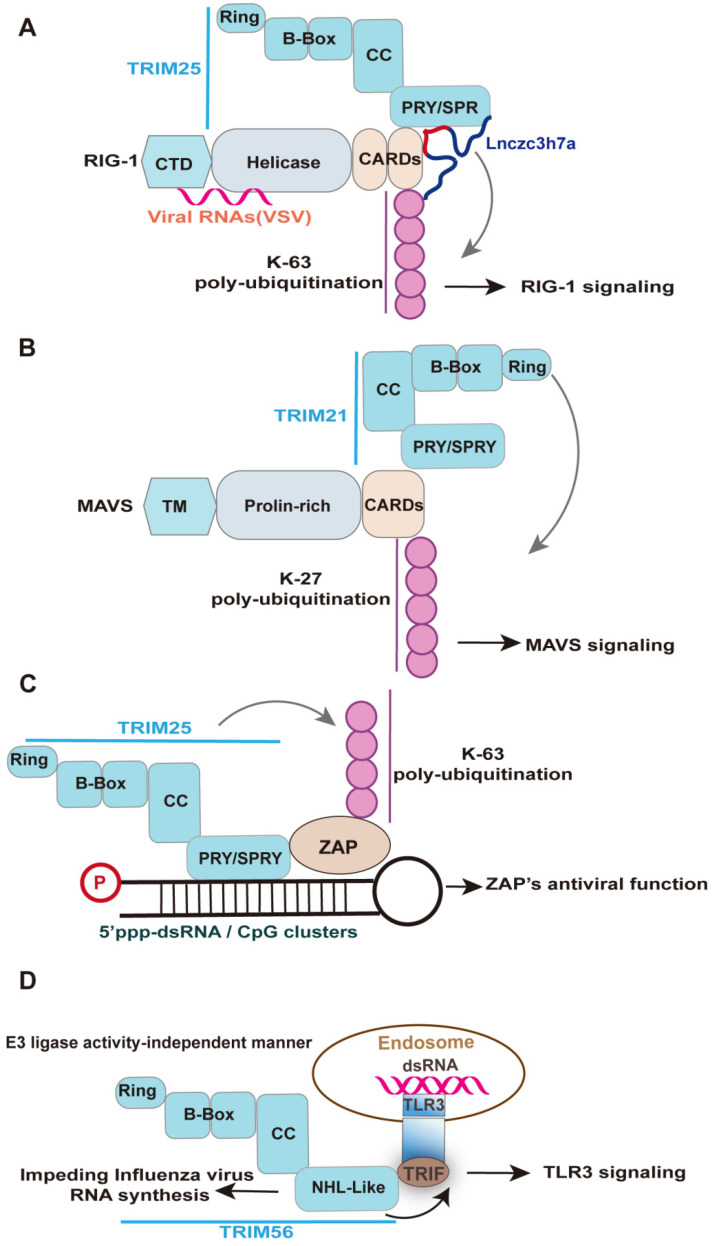
** Representative examples elucidating the role of RNA-binding domain of TRIM-family members in innate antiviral response.** Upon RNA virus infection, Lnczc3h7a is induced and binds to TRIM25 via SPRY domain and facilitates TRIM25-mediated K63-linked ubiquitination of RIG-I, promoting downstream signaling transduction of RIG-I. Under viral infection, TRIM21 interacts with MAVS and catalyzes its K27-linked polyubiquitination, thereby promoting MAVS-TBK1 signaling. Specifically, the PRY-SPRY domain of TRIM21 interacts with MAVS, while the RING domain of TRIM21 facilitates the K27-linked polyubiquitination of MAVS. TRIM25 is required for the antiviral function of ZAP. TRIM25 interacts with ZAP through its SPRY domain and mediates the K63-linked polyubiquitination of ZAP. Such modification enhances ZAP's antiviral activity, including viral RNA degradation, viral genes translation, and viral replication. Upon recognizing virus-derived dsRNA, the TLR3 adaptor TRIF forms a complex with TRIM56 by binding the NHL-like domain but not its full length, which is crucial for augmenting TLR3-mediated IFN response. Of note, the NHL-like domain of TRIM56 also specifically impede the intracellular influenza virus RNA synthesis, which whether involved in TRIM56-TRIF interaction remains unknown.

TRIM25 is a unique case of the TRIM-SPRY protein with RNA-binding activity required for its innate antiviral response 17, 137, 140. TRIM25 has been shown to bind both single and double-stranded RNAs, which are mainly attributed to the SPRY domain, a 7 Lysine peptide (7K), and the coiled-coil domain 17, 141. The RBDs of TRIM25 is crucial for auto-ubiquitinate itself and to ubiquitinate its target proteins RIG-I and ZAP 20, 21, 31, 141. Specifically, TRIM25 has been implicated in K63 ubiquitin activation of RIG-I antiviral signaling 17, 141, despite the apparent redundancy of TRIM25 in RIG-I-initiated IFN antiviral signaling with other E3 ubiquitin ligases 142, 143. The SPRY domain of TRIM25 interacts with the CARDs of RIG-I; this interaction effectively delivers the K63-linked ubiquitin moiety to the CARDs of RIG-I, resulting in activation of RIG-I signaling 31 (**Figure 4A**). Similarly, the ubiquitination of RIG-I two CARDs mediated by TRIM25 was significantly reduced in the TRIM25 7K mutant 141, which are in agreement with their effect on the virus replication 141. Moreover, a host lncRNA termed Lnczc3h7a binding the SPRY domain of TRIM25 enhances TRIM25-RIG-I interaction and RIG-I ubiquitination upon VSV infection, leading to an increased type I IFN response 20. In contrast to host lncRNA, the binding of the mutant sfRNA of Dengue virus clade (PR-2B) with a high affinity to TRIM25 reduces RIG-I signaling, leading to a decreased IFN-β expression 144. However, the increased ubiquitination of RIG-I would be an auto-ubiquitination of TRIM25 because more TRIM25 was co-immunoprecipitated with RIG-I in the presence of PR2B sfRNA 144. Indeed, a replacement of putative RNA-binding peptides within TRIM25 with the homologous sequences from other TRIM-PRY/SPRY proteins, including TRIM5α, TRIM25, TRIM27, TRIM21, and TRIM65, preserved the RNA binding activity 21, suggesting that functional parallel of TRIM-PRY/SPRY binding RNA. Indeed, TRIM21 is the crucial factor in enhancing type I IFN signaling 145, 146. The PRY-SPRY domain of TRIM21 interacts with MAVS, while the RING domain of TRIM21 facilitates the K27-linked polyubiquitination chains of MAVS 145 (**Figure 4B**). It would be interesting to investigate the role of lncRNA interactors of other TRIM-PRY/SPRY proteins, like its lncRNA interactor Lnczc3h7a, in the activation of downstream antiviral signaling 20. However, not every protein's RNA binding activity should be assumed to be physiologically or pathologically relevant. Indeed, the antivirus function mediated by the RBDs of TRIM family members did not always relate to RNA, such as TRIM14. TRIM14 interacts with the HBx protein of HBV via the SPRY domain and thereby inhibits viral replication 147.

TRIM25 also interacts with ZAP through its SPRY domain, with both the ubiquitin ligase activity and multimerization of TRIM25 enhancing ZAP's antiviral activity, including inhibition of virus translation, viral RNA degradation, and viral replication 21, 92, 148 (**Figure 4C**). Of note, despite the requirement of TRIM25 E3 ligase activity for enhancing ZAP-mediated inhibition of numerous virus the ubiquitination of ZAP itself did not directly affect antiviral activity against Sindbis virus 148. The importance of RNA binding of TRIM25 was supported by the complete abolition of poly-ubiquitination of TRIM25 and ZAP in the context of the RNase treatment 21. The RNA stress granules (SG) localization of TRIM25 is also mediated by its RNA binding activity 141. Indeed, RIG-I and ZAP are targeted to SG during viral infection, which is important for its antiviral activity 149, 150. Potentially, ZAP-TRIM25 or RIG-I-TRIM25 interaction may mediate the SG location of TRIM25.

The NHL domain is the earliest identified RBD among TRIM family members 137, 151, such as TRIM56 and TRIM71 138, 152, 153. TRIM56 is often not discussed in TRIM-NHL proteins but possesses NHL-like repeats domain 152, 154. The antiviral functions of TRIM56 mediated by the NHL-like domain were mainly thorough activating the TLR3 antiviral signaling pathway or inhibiting directly viral RNA synthesis 152, 153, 155 (**Figure 4D**), which depend on virus type. Specifically, a study from the Li group reported that TRIM56 via its NHL-like domain interacts with adaptor TRIF and thereby potentiates TLR3-mediated IRF3 activation and subsequent IFN response upon HCV infection 153. Their later study also demonstrated that the NHL-like domain of TRIM56 specifically impedes influenza virus RNA synthesis, but is ineffective in the inhibition of SeV, hMPV, and paramyxoviruses 152. In the case of the bovine diarrhoea virus, the entire C-terminus and the E3 ubiquitin ligase activity were essential for TRIM56 to restrict viral RNA replication 155. However, whether TRIM56 interacts with viral RNA remains unknown. Given influenza virus RNA synthesis occurs in the nucleus and IAV infection induced the nuclear translocation of the NHL-like domain of TRIM56 152, it may be possible that TRIM56 directly interacts with IAV RNA. Indeed, the inhibition effect of TRIM56 on viral RNA synthesis is virus-specific (above), which may be associated with the sequence of viral RNA, which enables them to be recognized by the NHL-like domain of TRIM56. The NHL domain of TRIM71 also binds host lncRNA to repress FGF/ERK signaling in embryonic stem cells, whereas its RNA binding function in innate antiviral response remains unknown^138^. Based on this perspective, the lncRNA interactors of TRIM56 may be crucial for the function of TRIM56.

### Roles of emerging RBPs in DNA sensors-initiated innate antiviral response

RBPs also function in DNA sensors-mediated innate immune response. The most typical examples are cGAS and its interacted RBPs. Indeed, despite as a traditionally recognized DNA binding protein, cGAS was recently found to be capable of binding a circular RNA named cia-cGAS in the nucleus 81. The binding of cia-cGAS to cGAS blocks the synthase activity of cGAS and thereby avoids the overproduction of type I IFNs to prevent long-term (LT) hematopoietic stem cells (HSCs) from exhaustion. Significantly, cia-cGAS showed a higher affinity with cGAS than self-DNA did in LT-HSCs 81, implicating the strong activity binding RNA of cGAS in type I IFNs although this study did not explore the cia-cGAS in innate antiviral response. Indeed, the interactions of cGAS with numerous RBPs play key roles in DNA-mediated innate antiviral response. GTPase-activating protein SH3 domain-binding protein 1 (G3BP1), a well-known RBP, is an interactor of cGAS for promoting DNA binding and activation of cGAS 125. G3BP1-mediated protein-RNA interactions network is the central node of the assemble of core SGs that are cytoplasmic foci enriched with RNAs and proteins when the cell is under stress 156-158. The assemble of SGs mediated by G3BP1 also regulates RIG-I-mediated innate antiviral response 159, 160, implying the importance of G3BP1 in the crosstalk of intracellular RNA- and DNA-sensing pathway. HEXIM1, another RBP interacting cGAS, corporates with NEAT1 to regulate the cGAS-mediated innate immune response in response to DNA virus KSHV 72. Further, cGAS-RBP interaction also functions in HIV-induced innate antiviral response, despite HIV is not a DNA virus. In detail, NONO is an RNA- and DNA-binding protein scaffold with numerous functions, including transcription, splicing, DNA damage response, and innate antiviral response 126, 161. Upon nuclear entry of HIV-2, the viral capsid can be detected by NONO and interacts with cGAS promoting its association with HIV-2 in the nucleus, enhancing cGAS-mediated activation of innate antiviral response 126. NONO also ensures the presence of cGAS in the nucleus, and that the chromatin state limits cGAS activation by self-DNA 126. However, the mechanism of cGAS nuclear translocation remains largely unknown. Given the RNA-binding activity of cGAS and its RBPs interactors, it would be a significant work of determining the roles of lncRNAs interactors of cGAS in innate antiviral response. Indeed, whether these RBPs can physiologically bind lncRNA and its functional importance in innate antiviral response remain unknown. In particular, whether RBDs or the RNAs binding RBDs mediate their function in innate antiviral response need to be further addressed by using the RNase to remove the RNA effect.

## Conclusion and future perspective

The role of lncRNAs and RBPs in innate antiviral response opened a new era of the regulation of host innate immunity and virus pathogenesis. Viral infection remarkably alters the expression profile of the host cell genome, including lncRNAs and RBPs 13, 18, 162-164. However, these differentially expressed genes were not equal to the functional factors in virus infection. The effect of lncRNAs on viral replication should be investigated using gain- or loss-of-function analysis to elucidate the essential role of lncRNA in the host-virus interaction. In particular, several lncRNAs (e.g. NEAT1) play divergent roles between innate antiviral response and viral gene expression, leading to different phenotypes of lncRNA intervention *in vitro* and *in vivo*. Therefore, the lncRNA-virus interaction would be more complicated than we expected. However, the effect of lncRNAs on viral infection should be better assessed *in vivo*, at which a joint effect would be observed. Indeed, lncRNAs usually do not show strict homology within model animals even some conserved lncRNAs undergo unconserved processing, localization, and function 6, 7, 165, posing challenges for their development and clinical application. Despite the robust methods for studying lncRNA 6, the surprisingly wide range of sizes, shapes, and functions of lncRNAs are still the challenges for their analysis. In particular, these characters partly conferred side effects to lncRNA-based drugs, which further hindered the research and development of lncRNAs. Identifying the conserved motifs that endow lncRNAs corresponding activity would be an efficient strategy for the development of nucleic acid-based drugs 166-168. Also, the selectively targeted delivery of lncRNA-based drugs would be a promising strategy to reduce its side effects 169. Currently, the clinical implication of lncRNAs is usually as biomarkers but not the lncRNA-based drugs 170, the latter of which is reported only in a few studies and still needs a long way to achieve. For instance, reducing UBE3A antisense transcript (UBE3A-ATS) with antisense oligonucleotides (ASOs) exhibited a potential therapeutic intervention for Angelman syndrome 171. Manipulation of lncRNA CCR5AS expression also affects HIV infection and disease progression 172. Besides, the phase 3 trial suggested that an RNA interference therapy Givosiran significantly reduced the rate of porphyria attacks and multiple other disease manifestations via inhibiting the expression of hepatic delta-aminolaevulinic acid synthase 1 (ALAS1) via a mechanism similar to lncRNA action 173.

Further, some of these lncRNAs induced by DNA virus would be recognized by RNA sensors to regulate innate antiviral response, implying the role of lncRNAs and RBPs in the crosstalk between DNA- and RNA-mediated innate antiviral response. Moreover, the lncRNAs induced by viral infection would be hijacked by the virus to escape host antiviral immune response, as a non-coding gene would work more efficiently than a coding-gene due to the lack of translation process. Indeed, the definition of one RBP should not be strictly defined by the classical RBDs as the mounting unconventional RBDs have been reported 16, 18, 19. From this perspective, the RNA that binds to the crucial factors in innate antiviral signaling may also participate in the regulation of innate antiviral response. Therefore, it would be significant for obtaining the lncRNA interactors of crucial components of type I IFNs signaling, although these factors were not typical RBPs. Indeed, prior large-scale RBP ChIP-seq analysis revealed widespread RBP presence in active chromatin regions in the human genome 174, implicating the importance of RBPs in the regulation of gene expression. Based on such a perspective, the DNA-binding proteins may also function as RBP by binding specific RNA, which needs to be explored in further research.

## Figures and Tables

**Table 1:** Roles of lncRNAs in innate antiviral response and the underlying mechanisms (ranked by the mechanisms of actions)

lncRNA	Classes	Species	Location	Mechanism of actions	Expression upon virus infection	Reference
Lnc-Lsm3b	Intronic	Mouse	Cytoplasm>>Nucleus	Inhibits I-IFNs production through binding RIG-I to restrict RIG-I proteins conformational shift	VSV, SeV; Upregulation	8
lncATV	Pseudogene	Human	Cytoplasm >> Nucleus	Inhibits the expression of type I IFNs through binding RIG-I to restrict RIG-I-mediated innate immunity	HCV, Zika virus, NDV, SeV; Upregulation	68
Lnczc3h7a	Intronic	Mouse	Cytoplasm>>Nucleus	Promotes a TRIM25-mediated RIG-I antiviral innate immune response	VSV, SeV; Upregulation	20
NEAT1	Intergenic	Human	Nucleus	Positively regulates the expression of IFN-β by promoting RIG-I and DDX60 expression	HTNV; Upregulation	69
ITPRIP-1	Intergenic	Human	Cytoplasm and nucleus	Positively regulates IFN signaling pathway through targeting MDA5	HCV, SeV, VSV, and HSV; Upregulation	70
NEAT1	Intergenic	Human	Nucleus	Positively regulates DNA-dependent activation of the cGAS-STING-IRF3 pathway	KSHV; N/A	71, 72
Lnc-ALVE1-AS1	Antisense	Endogenous retroviruses	Cytoplasm>>Nucleus	Induces antiviral response by activating the TLR3 signaling	ALVJ; N/A	73
lncLrrc55-AS	Antisense	Mouse and Human	Cytoplasm>>Nucleus	Promotes I-IFNs signaling by strengthening IRF3 phosphorylation	SeV, HSV-1, VSV, IAV; Upregulation	74
#32	Antisense	Human	N/A	Positively regulate the expression of ISGs by binding to ATF2	EMCV, HBV, HCV; Downregulation	75
lncRNA-155	N/A	Mouse and Human	Nucleus >> Cytoplasm	Inhibits the expression of PTP1B and thereby activates TYK2-JAK2 signaling to facilitate the expression of ISGs	IAV, MDRV, SeV; Upregulation	76
NRAV	Antisense	Human	Nucleus>>Cytoplasm	Negatively regulates the expression of IFITM3 and MxA by affecting histone modification of these genes	IAV, SeV, MDRV, HSV; Downregulation	10
IVRPIE	Promoter	Human	Nucleus >> Cytoplasm	Promotes the expression of IFN-β and ISGs by modifying their promoter activity through an interaction with hnRNPU	IAV, SeV, VSIV, VSNJV; Upregulation	77
EGOT	Intronic	Human	Nucleus>>Cytoplasm	Negatively regulates the expression of ISGs with an unknown mechanism	HCV, SFV, IAV; Upregulation	78
Lnc-ITM2C-1	Intergenic	Human	Nucleus>>Cytoplasm	Negatively regulates the expression of ISGs by stimulating expression of GPR55	HCV; Upregulation	79
lncRNA-CMPK2	Intergenic	Human	Nucleus	Negatively regulates the transcription of IFN-stimulated antiviral genes with unknown mechanism	HCV; Upregulation	80

ATF2, activating transcription factor 2; SFV, Semliki Forest virus; CEFs, chicken embryonic fibroblasts; ALVJ, avian leukosis virus subgroup J; NDV, Newcastle disease virus; SeV, Sendai virus; GPR55, G protein-coupled receptor 55; VSNJV, VSV New Jersey; VSIV, VSV Indiana; RSV, Respiratory Syncytial Virus; hnRNPU, heterogeneous nuclear ribonuclear protein U; TLR3, Toll-like receptor 3. N/A, not applicable.

**Table 2:** Roles of RBPs in innate antiviral response

Name	Species	Virus	RNA interactors	Mechanism of action	Protein interactors	Reference
TRIM25	Human and Mouse	SeV, IAV, EMCV	Lnczc3h7a(in mice)	Mediates K63-linked poly-ubiquitination of the RIG-I	RIG-I	20, 31
PACT	Human and Mouse	EMCV, SeV, TMEV, HSV-1	N/A	Enhances MDA5- and RIG-I-mediated immune responses;	LGP2, Us11, and RIG-I	117-119
4a	MERS-CoV	MERS-CoV	N/A	Suppresses PACT-induced activation of RIG-I and MDA5 in the innate antiviral response	PACT*	120
FTSJ3	Human	HIV	HIV RNA	FTSJ3 can be recruited by TRBP to enhance the 2'-O-methylations of HIV RNA to avoid MDA5-mediated antiviral immune response	TRBP	116
STAU1	Chicken	IBDV	Viral genomic dsRNA	Attenuates MDA5-mediated induction of IFN-β	N/A	121
PUM1	Human	HSV-1	N/A	Negative regulator of innate immunity genes by suppressing LGP2	N/A	122
HuR	Human and mouse	NDV	*PLK2* mRNA	Bolsters RLR-mediated IRF3 nuclear translocation by controlling the stability of Plk2 mRNA; Maintains the stability of *Ifnb1* mRNA	N/A	123, 124
PCBP2	Human	VSV, SeV, NDV, HCV	N/A	Mediates the degradation of MAVS via the E3 ubiquitin ligase AIP4 or NLRX1	MAVS, RIG-I, MDA5, and AIP4	97, 98
PCBP1	Human	SeV, NDV, VSV	N/A	Mediates the housekeeping degradation of MAVS	Above	96
hnRNPA2B1	Human and Mouse	HSV-1	N/A	Initiates and amplifies the innate immune response to DNA viruses	TBK1, JMJD6	48
G3BP1	Human and Mouse	HSV-1	N/A	Promotes DNA binding and activation of cGAS	cGAS	125
NONO	Human	HIV-1 and HIV-2	N/A	NONO is essential for cGAS activation by HIV and cGAS association with HIV DNA in the nucleus	cGAS	126
TRIM14	Human and Mouse	HSV-1	N/A	Inhibits cGAS degradation mediated by selective autophagy receptor p62	cGAS, p62, USP14	127
HEXIM1	Human	KSHV	NEAT1	Positively regulates DNA-dependent activation of the cGAS-STING-IRF3 pathway	DNA-PK, SFPQ, PSPC1, and NONO	72
TRIM27	Mouse	VSV, SeV, HSV-1	N/A	Induces TBK1 degradation	DAP12, SHP2, TBK1	128
Roquin	Human	HCMV	*IRF1* mRNA	Reduces IRF1 expression by directly binding to its mRNA	N/A	129, 130
TRBP	Human	HIV	N/A	Support HIV-1 infection by inhibiting PKR-mediated Antiviral Response	IFIT3	131
IFIT1	Human	WNV and ZIKV	Viral RNA	Binds to viral cap 0 RNA to restrict viral genes translation	N/A	132
TRIM56	Human	ZIKV	ZIKV RNA	Restricts ZIKV replication through binding ZIKV RNA	N/A	133
IRAV	Human	EMCV, VSV, DENV	N/A	Associates with P-bodies within the viral replication compartments	MOV10	134
ORF57	KSHV	KSHV	N/A	Inhibits P-bodies formation to promote viral replication by an interaction with Ago2 and GW182.	Ago2, GW182	135
DBR1	Human	HSV-1, IAV, NV	N/A	Confers the resistance of CNS against virus infection by maintaining the RNA lariat metabolism	N/A	22

## Abbreviations

lncRNA: long non-coding RNA
RBP: RNA-binding proteins
ATF2: activating transcription factor 2
SFV: Semliki Forest virus
CEFs: chicken embryonic fibroblasts
ALVJ: avian leukosis virus subgroup J
NDV: Newcastle disease virus
SeV: Sendai virus
GPR55: G protein-coupled receptor 55
VSNJV: VSV New Jersey
VSIV: VSV Indiana
RSV: Respiratory Syncytial Virus
hnRNPU: heterogeneous nuclear ribonuclear protein U
TLR3: Toll-like receptor 3
TRIM25: tripartite motif containing 25
PACT: protein activator of interferon induced protein kinase EIF2AK2
4a: Middle east respiratory syndrome coronavirus 4a protein
FTSJ3: FtsJ RNA 2'-O-Methyltransferase 3
STAU1: staufen double-stranded RNA binding protein 1
PUM1: pumilio RNA binding family member 1
HuR: Hu antigen R
PCBP2: poly(RC) binding protein 2
PCBP1: poly(RC) binding protein 1
hnRNPA2B1: heterogeneous nuclear ribonucleoprotein A2/B1
G3BP1: G3BP stress granule assembly factor 1
NONO: non-POU domain containing octamer binding
TRIM14: tripartite motif containing 14
HEXIM1: HEXIM P-TEFb complex subunit 1
TRIM27: tripartite motif containing 27
TRBP: TAR RNA binding protein
IFIT1: interferon induced protein with tetratricopeptide repeats 1
TRIM56: tripartite motif containing 14
IRAV: interferon regulated antiviral gene
ORF57: KSHV RNA-binding protein ORF57
NAT: natural antisense transcript

## References

[1] Blanco-Melo D, Nilsson-Payant BE, Liu W-C, Uhl S, Hoagland D, Møller R (2020). Imbalanced Host Response to SARS-CoV-2 Drives Development of COVID-19. Cell.

[2] Wang Y, Jia J, Wang Y, Li F, Song X, Qin S (2019). Roles of HSV-1 infection-induced microglial immune responses in CNS diseases: friends or foes?. Crit Rev Microbiol.

[3] Hajishengallis G, Lambris JD (2011). Microbial manipulation of receptor crosstalk in innate immunity. Nat Rev Immunol.

[4] Ivashkiv LB, Donlin LT (2014). Regulation of type I interferon responses. Nat Rev Immunol.

[5] Ma Z, Damania B (2016). The cGAS-STING Defense Pathway and Its Counteraction by Viruses. Cell Host Microbe.

[6] Yao RW, Wang Y, Chen LL (2019). Cellular functions of long noncoding RNAs. Nat Cell Biol.

[7] Zhang Y, Cao X (2016). Long noncoding RNAs in innate immunity. Cell Mol Immunol.

[8] Jiang M, Zhang S, Yang Z, Lin H, Zhu J, Liu L (2018). Self-Recognition of an Inducible Host lncRNA by RIG-I Feedback Restricts Innate Immune Response. Cell.

[9] Pan Q, Zhao Z, Liao Y, Chiu SH, Wang S, Chen B (2019). Identification of an Interferon-Stimulated Long Noncoding RNA (LncRNA ISR) Involved in Regulation of Influenza A Virus Replication. Int J Mol Sci.

[10] Ouyang J, Zhu X, Chen Y, Wei H, Chen Q, Chi X (2014). NRAV, a long noncoding RNA, modulates antiviral responses through suppression of interferon-stimulated gene transcription. Cell Host Microbe.

[11] Ouyang J, Hu J, Chen JL (2016). lncRNAs regulate the innate immune response to viral infection. WIREs RNA.

[12] Qiu L, Wang T, Tang Q, Li G, Wu P, Chen K (2018). Long Non-coding RNAs: Regulators of Viral Infection and the Interferon Antiviral Response. Front Microbiol.

[13] Feng J, Yang G, Liu Y, Gao Y, Zhao M, Bu Y (2019). LncRNA PCNAP1 modulates hepatitis B virus replication and enhances tumor growth of liver cancer. Theranostics.

[14] Peng X, Gralinski L, Armour CD, Ferris MT, Thomas MJ, Proll S (2010). Unique Signatures of Long Noncoding RNA Expression in Response to Virus Infection and Altered Innate Immune Signaling. mBio.

[15] Ma L, Bajic VB, Zhang Z (2013). On the classification of long non-coding RNAs. RNA Biol.

[16] Li JH, Liu S, Zheng LL, Wu J, Sun WJ, Wang ZL (2014). Discovery of Protein-lncRNA Interactions by Integrating Large-Scale CLIP-Seq and RNA-Seq Datasets. Front Bioeng Biotechnol.

[17] Choudhury NR, Heikel G, Michlewski G (2020). TRIM25 and its emerging RNA-binding roles in antiviral defense. WIREs RNA.

[18] Garcia-Moreno M, Jarvelin AI, Castello A (2018). Unconventional RNA-binding proteins step into the virus-host battlefront. WIREs RNA.

[19] Hentze MW, Castello A, Schwarzl T, Preiss T (2018). A brave new world of RNA-binding proteins. Nat Rev Mol Cell Biol.

[20] Lin H, Jiang M, Liu L, Yang Z, Ma Z, Liu S (2019). The long noncoding RNA Lnczc3h7a promotes a TRIM25-mediated RIG-I antiviral innate immune response. Nat Immunol.

[21] Choudhury NR, Heikel G, Trubitsyna M, Kubik P, Nowak JS, Webb S (2017). RNA-binding activity of TRIM25 is mediated by its PRY/SPRY domain and is required for ubiquitination. BMC Biol.

[22] Zhang SY, Clark NE, Freije CA, Pauwels E, Taggart AJ, Okada S (2018). Inborn Errors of RNA Lariat Metabolism in Humans with Brainstem Viral Infection. Cell.

[23] Lafaille FG, Harschnitz O, Lee YS, Zhang P, Hasek ML, Kerner G (2019). Human SNORA31 variations impair cortical neuron-intrinsic immunity to HSV-1 and underlie herpes simplex encephalitis. Nat Med.

[24] Brubaker SW, Bonham KS, Zanoni I, Kagan JC (2015). Innate immune pattern recognition: a cell biological perspective. Annu Rev Immunol.

[25] Takeuchi O, Akira S (2010). Pattern recognition receptors and inflammation. Cell.

[26] Kawai T, Akira S (2009). The roles of TLRs, RLRs and NLRs in pathogen recognition. Int Immunol.

[27] Yoneyama M, Kikuchi M, Natsukawa T, Shinobu N, Imaizumi T, Miyagishi M (2004). The RNA helicase RIG-I has an essential function in double-stranded RNA-induced innate antiviral responses. Nat Immunol.

[28] Wu B, Peisley A, Richards C, Yao H, Zeng X, Lin C (2013). Structural basis for dsRNA recognition, filament formation, and antiviral signal activation by MDA5. Cell.

[29] Yoneyama M, Kikuchi M, Matsumoto K, Imaizumi T, Miyagishi M, Taira K (2005). Shared and unique functions of the DExD/H-box helicases RIG-I, MDA5, and LGP2 in antiviral innate immunity. J Immunol.

[30] Chiu YH, Macmillan JB, Chen ZJ (2009). RNA polymerase III detects cytosolic DNA and induces type I interferons through the RIG-I pathway. Cell.

[31] Gack MU, Shin YC, Joo C-H, Urano T, Liang C, Sun L (2007). TRIM25 RING-finger E3 ubiquitin ligase is essential for RIG-I-mediated antiviral activity. Nature.

[32] Oshiumi H, Matsumoto M, Hatakeyama S, Seya T (2008). Riplet/RNF135, a RING Finger Protein, Ubiquitinates RIG-I to Promote Interferon-beta Induction during the Early Phase of Viral Infection. J BIOL CHEM.

[33] Oshiumi H, Miyashita M, Inoue N, Okabe M, Matsumoto M, Seya T (2010). The Ubiquitin Ligase Riplet Is Essential for RIG-I-Dependent Innate Immune Responses to RNA Virus Infection. Cell host & microbe.

[34] Liu Helene M, Loo Y-M, Horner Stacy M, Zornetzer Gregory A, Katze Michael G, Gale M (2012). The Mitochondrial Targeting Chaperone 14-3-3ε Regulates a RIG-I Translocon that Mediates Membrane Association and Innate Antiviral Immunity. Cell Host & Microbe.

[35] Seth RB, Sun L, Ea CK, Chen ZJ (2005). Identification and characterization of MAVS, a mitochondrial antiviral signaling protein that activates NF-kappaB and IRF 3. Cell.

[36] Dixit E, Boulant S, Zhang Y, Lee A, Odendall C, Shum B (2010). Peroxisomes Are Signaling Platforms for Antiviral Innate Immunity. Cell.

[37] Horner SM, Liu HM, Park HS, Briley J, Gale M (2011). Mitochondrial-associated endoplasmic reticulum membranes (MAM) form innate immune synapses and are targeted by hepatitis C virus. Proc Natl Acad Sci USA.

[38] Hou F, Sun L, Zheng H, Skaug B, Jiang QX, Chen ZJ (2011). MAVS forms functional prion-like aggregates to activate and propagate antiviral innate immune response. Cell.

[39] Liu S, Chen J, Cai X, Wu J, Chen X, Wu Y-T (2013). MAVS recruits multiple ubiquitin E3 ligases to activate antiviral signaling cascades. eLife.

[40] Alexopoulou L, Czopik A, Medzhitov R, Flavell R (2001). Recognition of double-stranded RNA and activation of NF-B by Toll-like receptor 3. Nature.

[41] Guo Y, Audry M, Ciancanelli M, Alsina L, Azevedo J, Herman M (2011). Herpes simplex virus encephalitis in a patient with complete TLR3 deficiency: TLR3 is otherwise redundant in protective immunity. J Exp Med.

[42] Zhang SY, Jouanguy E, Ugolini S, Smahi A, Elain G, Romero P (2007). TLR3 deficiency in patients with herpes simplex encephalitis. Science.

[43] Ishikawa H, Ma Z, Barber GN (2009). STING regulates intracellular DNA-mediated, type I interferon-dependent innate immunity. Nature.

[44] Sun L, Wu J, Du F, Chen X, Chen ZJ (2013). Cyclic GMP-AMP synthase is a cytosolic DNA sensor that activates the type I interferon pathway. Science.

[45] Wu J, Sun L, Chen X, Du F, Shi H, Chen C (2012). Cyclic GMP-AMP Is an Endogenous Second Messenger in Innate Immune Signaling by Cytosolic DNA. Science.

[46] Saitoh T, Fujita N, Hayashi T, Takahara K, Satoh T, Lee H (2009). Atg9a controls dsDNA-driven dynamic translocation of STING and the innate immune response. Proc Natl Acad Sci U S A.

[47] Zhang Z, Yuan B, Bao M, Lu N, Kim T, Liu YJ (2011). The helicase DDX41 senses intracellular DNA mediated by the adaptor STING in dendritic cells. Nat Immunol.

[48] Wang L, Wen M, Cao X (2019). Nuclear hnRNPA2B1 initiates and amplifies the innate immune response to DNA viruses. Science.

[49] Hemmi H, Takeuchi O, Kawai T, Kaisho T, Sato S, Sanjo H (2000). A Toll-like receptor recognizes bacterial DNA. Nature.

[50] Honda K, Yanai H, Negishi H, Asagiri M, Sato M, Mizutani T (2005). IRF-7 is the master regulator of type-I interferon-dependent immune responses. Nature.

[51] Fernandes-Alnemri T, Yu JW, Datta P, Wu J, Alnemri ES (2009). AIM2 activates the inflammasome and cell death in response to cytoplasmic DNA. Nature.

[52] Hornung V, Ablasser A, Charrel-Dennis M, Bauernfeind F, Horvath G, Caffrey DR (2009). AIM2 recognizes cytosolic dsDNA and forms a caspase-1-activating inflammasome with ASC. Nature.

[53] Burckstummer T, Baumann C, Bluml S, Dixit E, Durnberger G, Jahn H (2009). An orthogonal proteomic-genomic screen identifies AIM2 as a cytoplasmic DNA sensor for the inflammasome. Nat Immunol.

[54] Lamkanfi M, Dixit VM (2014). Mechanisms and functions of inflammasomes. Cell.

[55] Gray EE, Winship D, Snyder JM, Child SJ, Geballe AP, Stetson DB (2016). The AIM2-like Receptors Are Dispensable for the Interferon Response to Intracellular DNA. Immunity.

[56] Levy DE, Darnell JE Jr (2002). Stats: transcriptional control and biological impact. Nat Rev Mol Cell Biol.

[57] Stark GR, Darnell JE Jr (2012). The JAK-STAT pathway at twenty. Immunity.

[58] MacMicking JD (2012). Interferon-inducible effector mechanisms in cell-autonomous immunity. Nat Rev Immunol.

[59] Saka HA, Valdivia R (2012). Emerging roles for lipid droplets in immunity and host-pathogen interactions. Annu Rev Cell Dev Biol.

[60] Lee AJ, Ashkar AA (2018). The Dual Nature of Type I and Type II Interferons. Front Immunol.

[61] Chen K, Liu J, Cao X (2017). Regulation of type I interferon signaling in immunity and inflammation: A comprehensive review. J Autoimmun.

[62] Buchmeier MJ, Sommereyns C, Paul S, Staeheli P, Michiels T (2008). IFN-Lambda (IFN-λ) Is Expressed in a Tissue-Dependent Fashion and Primarily Acts on Epithelial Cells *In Vivo*. PLOS Pathog.

[63] Ank N, Iversen MB, Bartholdy C, Staeheli P, Hartmann R, Jensen UB (2008). An important role for type III interferon (IFN-lambda/IL-28) in TLR-induced antiviral activity. J Immunol.

[64] Ank N, West H, Bartholdy C, Eriksson K, Thomsen AR, Paludan SR (2006). Lambda Interferon (IFN-λ), a Type III IFN, Is Induced by Viruses and IFNs and Displays Potent Antiviral Activity against Select Virus Infections *In Vivo*. J Virol.

[65] Zhou Z, Hamming OJ, Ank N, Paludan SR, Nielsen AL, Hartmann R (2007). Type III Interferon (IFN) Induces a Type I IFN-Like Response in a Restricted Subset of Cells through Signaling Pathways Involving both the Jak-STAT Pathway and the Mitogen-Activated Protein Kinases. J Virol.

[66] McNab F, Mayer-Barber K, Sher A, Wack A, O'Garra A (2015). Type I interferons in infectious disease. Nat Rev Immunol.

[67] Stetson DB, Medzhitov R (2006). Type I interferons in host defense. Immunity.

[68] Fan J, Cheng M, Chi X, Liu X, Yang W (2019). A Human Long Non-coding RNA LncATV Promotes Virus Replication Through Restricting RIG-I-Mediated Innate Immunity. Front Immunol.

[69] Ma H, Han P, Ye W, Chen H, Zheng X, Cheng L (2017). The Long Noncoding RNA NEAT1 Exerts Antihantaviral Effects by Acting as Positive Feedback for RIG-I Signaling. J Virol.

[70] Xie Q, Chen S, Tian R, Huang X, Deng R, Xue B (2018). Long Noncoding RNA ITPRIP-1 Positively Regulates the Innate Immune Response through Promotion of Oligomerization and Activation of MDA5. J Virol.

[71] Hutchinson JN, Ensminger AW, Clemson CM, Lynch CR, Lawrence JB, Chess A (2007). A screen for nuclear transcripts identifies two linked noncoding RNAs associated with SC35 splicing domains. BMC Genom.

[72] Morchikh M, Cribier A, Raffel R, Amraoui S, Cau J, Severac D (2017). HEXIM1 and NEAT1 Long Non-coding RNA Form a Multi-subunit Complex that Regulates DNA-Mediated Innate Immune Response. Mol Cell.

[73] Chen S, Hu X, Cui IH, Wu S, Dou C, Liu Y (2019). An endogenous retroviral element exerts an antiviral innate immune function via the derived lncRNA lnc-ALVE1-AS1. Antiviral Res.

[74] Zhou Y, Li M, Xue Y, Li Z, Wen W, Liu X (2019). Interferon-inducible cytoplasmic lncLrrc55-AS promotes antiviral innate responses by strengthening IRF3 phosphorylation. Cell Research.

[75] Nishitsuji H, Ujino S, Yoshio S, Sugiyama M, Mizokami M, Kanto T (2016). Long noncoding RNA #32 contributes to antiviral responses by controlling interferon-stimulated gene expression. Proc Natl Acad Sci U S A.

[76] Maarouf M, Chen B, Chen Y, Wang X, Rai KR, Zhao Z (2019). Identification of lncRNA-155 encoded by MIR155HG as a novel regulator of innate immunity against influenza A virus infection. Cell Microbiol.

[77] Zhao L, Xia M, Wang K, Lai C, Fan H, Gu H (2020). A Long Non-coding RNA IVRPIE Promotes Host Antiviral Immune Responses Through Regulating Interferon beta1 and ISG Expression. Front Microbiol.

[78] Carnero E, Barriocanal M, Prior C, Pablo Unfried J, Segura V, Guruceaga E (2016). Long noncoding RNA EGOT negatively affects the antiviral response and favors HCV replication. EMBO Rep.

[79] Hu P, Wilhelm J, Gerresheim G, Shalamova L, Niepmann M (2019). Lnc-ITM2C-1 and GPR55 Are Proviral Host Factors for Hepatitis C Virus. Viruses.

[80] Kambara H, Niazi F, Kostadinova L, Moonka DK, Siegel CT, Post AB (2014). Negative regulation of the interferon response by an interferon-induced long non-coding RNA. Nucleic Acids Res.

[81] Xia P, Wang S, Ye B, Du Y, Li C, Xiong Z (2018). A Circular RNA Protects Dormant Hematopoietic Stem Cells from DNA Sensor cGAS-Mediated Exhaustion. Immunity.

[82] Imamura K, Imamachi N, Akizuki G, Kumakura M, Kawaguchi A, Nagata K (2014). Long noncoding RNA NEAT1-dependent SFPQ relocation from promoter region to paraspeckle mediates IL8 expression upon immune stimuli. Mol Cell.

[83] Li W, Notani D, Rosenfeld MG (2016). Enhancers as non-coding RNA transcription units: recent insights and future perspectives. Nat Rev Genet.

[84] Poliseno L, Salmena L, Zhang J, Carver B, Haveman WJ, Pandolfi PP (2010). A coding-independent function of gene and pseudogene mRNAs regulates tumour biology. Nature.

[85] Salmena L, Poliseno L, Tay Y, Kats L, Pandolfi PP (2011). A ceRNA hypothesis: the Rosetta Stone of a hidden RNA language?. Cell.

[86] Tormanen K, Allen S, Mott KR, Ghiasi H (2019). The Latency-Associated Transcript Inhibits Apoptosis via Downregulation of Components of the Type I Interferon Pathway during Latent Herpes Simplex Virus 1 Ocular Infection. J Virol.

[87] Phelan D, Barrozo ER, Bloom DC (2017). HSV1 latent transcription and non-coding RNA: A critical retrospective. J Neuroimmunol.

[88] Wang P, Xue Y, Han Y, Lin L, Wu C, Xu S (2014). The STAT3-binding long noncoding RNA lnc-DC controls human dendritic cell differentiation. Science.

[89] Carpenter S, Aiello D, Atianand MK, Ricci EP, Gandhi P, Hall LL (2013). A Long Noncoding RNA Mediates Both Activation and Repression of Immune Response Genes. Science.

[90] Li Z, Chao TC, Chang KY, Lin N, Patil VS, Shimizu C (2014). The long noncoding RNA THRIL regulates TNFalpha expression through its interaction with hnRNPL. Proc Natl Acad Sci U S A.

[91] Luo X, Wang X, Gao Y, Zhu J, Liu S, Gao G (2020). Molecular Mechanism of RNA Recognition by Zinc-Finger Antiviral Protein. Cell Rep.

[92] Takata MA, Goncalves-Carneiro D, Zang TM, Soll SJ, York A, Blanco-Melo D (2017). CG dinucleotide suppression enables antiviral defence targeting non-self RNA. Nature.

[93] Schwerk J, Soveg FW, Ryan AP, Thomas KR, Hatfield LD, Ozarkar S (2019). RNA-binding protein isoforms ZAP-S and ZAP-L have distinct antiviral and immune resolution functions. Nat Immunol.

[94] Guo X, Carroll JW, Macdonald MR, Goff SP, Gao G (2004). The zinc finger antiviral protein directly binds to specific viral mRNAs through the CCCH zinc finger motifs. J Virol.

[95] Zhu Y, Wang X, Goff SP, Gao G (2012). Translational repression precedes and is required for ZAP-mediated mRNA decay. EMBO J.

[96] Zhou X, You F, Chen H, Jiang Z (2012). Poly(C)-binding protein 1 (PCBP1) mediates housekeeping degradation of mitochondrial antiviral signaling (MAVS). Cell Res.

[97] You F, Sun H, Zhou X, Sun W, Liang S, Zhai Z (2009). PCBP2 mediates degradation of the adaptor MAVS via the HECT ubiquitin ligase AIP4. Nat Immunol.

[98] Qin Y, Xue B, Liu C, Wang X, Tian R, Xie Q (2017). NLRX1 Mediates MAVS Degradation To Attenuate the Hepatitis C Virus-Induced Innate Immune Response through PCBP2. J Virol.

[99] Ferre F, Colantoni A, Helmer-Citterich M (2016). Revealing protein-lncRNA interaction. Brief Bioinform.

[100] Hu H, Zhu C, Ai H, Zhang L, Zhao J, Zhao Q (2017). LPI-ETSLP: lncRNA-protein interaction prediction using eigenvalue transformation-based semi-supervised link prediction. Mol Biosyst.

[101] Zhao Q, Zhang Y, Hu H, Ren G, Zhang W, Liu H (2018). IRWNRLPI: Integrating Random Walk and Neighborhood Regularized Logistic Matrix Factorization for lncRNA-Protein Interaction Prediction. Front Genet.

[102] Albihlal WS, Gerber AP (2018). Unconventional RNA-binding proteins: an uncharted zone in RNA biology. FEBS Lett.

[103] Kino T, Hurt DE, Ichijo T, Nader N, Chrousos GP (2010). Noncoding RNA Gas5 Is a Growth Arrest- and Starvation-Associated Repressor of the Glucocorticoid Receptor. Sci Signal.

[104] Tani H, Torimura M, Akimitsu N (2013). The RNA degradation pathway regulates the function of GAS5 a non-coding RNA in mammalian cells. PLoS One.

[105] Huang H, Weng H, Chen J (2020). m(6)A Modification in Coding and Non-coding RNAs: Roles and Therapeutic Implications in Cancer. Cancer Cell.

[106] Desrosiers R, Friderici K, Rottman F (1974). Identification of Methylated Nucleosides in Messenger RNA from Novikoff Hepatoma Cells. Proc Natl Acad Sci USA.

[107] Shulman Z, Stern-Ginossar N (2020). The RNA modification N6-methyladenosine as a novel regulator of the immune system. Nat Immunol.

[108] Frye M, Harada BT, Behm M, He C (2018). RNA modifications modulate gene expression during development. Science.

[109] Li X-C, Jin F, Wang B-Y, Yin X-J, Hong W, Tian F-J (2019). The m6A demethylase ALKBH5 controls trophoblast invasion at the maternal-fetal interface by regulating the stability of CYR61 mRNA. Theranostics.

[110] Zheng Q, Hou J, Zhou Y, Li Z, Cao X (2017). The RNA helicase DDX46 inhibits innate immunity by entrapping m(6)A-demethylated antiviral transcripts in the nucleus. Nat Immunol.

[111] Winkler R, Gillis E, Lasman L, Safra M, Geula S, Soyris C (2019). m(6)A modification controls the innate immune response to infection by targeting type I interferons. Nat Immunol.

[112] Rubio RM, Depledge DP, Bianco C, Thompson L, Mohr I (2018). RNA m(6) A modification enzymes shape innate responses to DNA by regulating interferon beta. Genes Dev.

[113] Zhang Y, Wang X, Zhang X, Wang J, Ma Y, Zhang L (2019). RNA-binding protein YTHDF3 suppresses interferon-dependent antiviral responses by promoting FOXO3 translation. Proc Natl Acad Sci U S A.

[114] Litvak V, Ratushny AV, Lampano AE, Schmitz F, Huang AC, Raman A (2012). A FOXO3-IRF7 gene regulatory circuit limits inflammatory sequelae of antiviral responses. Nature.

[115] Liu Y, You Y, Lu Z, Yang J, Li P, Liu L (2019). N (6)-methyladenosine RNA modification-mediated cellular metabolism rewiring inhibits viral replication. Science.

[116] Ringeard M, Marchand V, Decroly E, Motorin Y, Bennasser Y (2019). FTSJ3 is an RNA 2'-O-methyltransferase recruited by HIV to avoid innate immune sensing. Nature.

[117] Miyamoto M, Komuro A (2017). PACT is required for MDA5-mediated immunoresponses triggered by Cardiovirus infection via interaction with LGP2. Biochem Biophys Res Commun.

[118] Kew C, Lui PY, Chan CP, Liu X, Au SW, Mohr I (2013). Suppression of PACT-induced type I interferon production by herpes simplex virus 1 Us11 protein. J Virol.

[119] Kok KH, Lui PY, Ng MH, Siu KL, Au SW, Jin DY (2011). The double-stranded RNA-binding protein PACT functions as a cellular activator of RIG-I to facilitate innate antiviral response. Cell Host Microbe.

[120] Siu KL, Yeung ML, Kok KH, Yuen KS, Kew C, Lui PY (2014). Middle east respiratory syndrome coronavirus 4a protein is a double-stranded RNA-binding protein that suppresses PACT-induced activation of RIG-I and MDA5 in the innate antiviral response. J Virol.

[121] Ye C, Yu Z, Xiong Y, Wang Y, Ruan Y, Guo Y (2019). STAU1 binds to IBDV genomic double-stranded RNA and promotes viral replication via attenuation of MDA5-dependent beta interferon induction. FASEB J.

[122] Liu Y, Qu L, Liu Y, Roizman B, Zhou GG (2017). PUM1 is a biphasic negative regulator of innate immunity genes by suppressing LGP2. Proc Natl Acad Sci U S A.

[123] Sueyoshi T, Kawasaki T, Kitai Y, Ori D, Akira S, Kawai T (2018). Hu Antigen R Regulates Antiviral Innate Immune Responses through the Stabilization of mRNA for Polo-like Kinase 2. J Immunol.

[124] Takeuchi O (2015). HuR keeps interferon-beta mRNA stable. Eur J Immunol.

[125] Liu ZS, Cai H, Xue W, Wang M, Xia T, Li WJ (2019). G3BP1 promotes DNA binding and activation of cGAS. Nat Immunol.

[126] Lahaye X, Gentili M, Silvin A, Conrad C, Picard L, Jouve M (2018). NONO Detects the Nuclear HIV Capsid to Promote cGAS-Mediated Innate Immune Activation. Cell.

[127] Chen M, Meng Q, Qin Y, Liang P, Tan P, He L (2016). TRIM14 Inhibits cGAS Degradation Mediated by Selective Autophagy Receptor p62 to Promote Innate Immune Responses. Mol Cell.

[128] Zheng Q, Hou J, Zhou Y, Yang Y, Xie B, Cao X (2015). Siglec1 suppresses antiviral innate immune response by inducing TBK1 degradation via the ubiquitin ligase TRIM27. Cell Res.

[129] Song J, Lee S, Cho DY, Lee S, Kim H, Yu N (2019). Human cytomegalovirus induces and exploits Roquin to counteract the IRF1-mediated antiviral state. Proc Natl Acad Sci U S A.

[130] Athanasopoulos V, Ramiscal RR, Vinuesa CG (2016). ROQUIN signalling pathways in innate and adaptive immunity. Eur J Immunol.

[131] Ong CL, Thorpe JC, Gorry PR, Bannwarth S, Jaworowski A, Howard JL (2005). Low TRBP levels support an innate human immunodeficiency virus type 1 resistance in astrocytes by enhancing the PKR antiviral response. J Virol.

[132] Johnson B, VanBlargan LA, Xu W, White JP, Shan C, Shi PY (2018). Human IFIT3 Modulates IFIT1 RNA Binding Specificity and Protein Stability. Immunity.

[133] Yang D, Li NL, Wei D, Liu B, Guo F, Elbahesh H (2019). The E3 ligase TRIM56 is a host restriction factor of Zika virus and depends on its RNA-binding activity but not miRNA regulation, for antiviral function. PLoS Negl Trop Dis.

[134] Balinsky CA, Schmeisser H, Wells AI, Ganesan S, Jin T, Singh K (2017). IRAV (FLJ11286), an Interferon-Stimulated Gene with Antiviral Activity against Dengue Virus, Interacts with MOV10. J Virol.

[135] Sharma NR, Majerciak V, Kruhlak MJ, Yu L, Kang JG, Yang A (2019). KSHV RNA-binding protein ORF57 inhibits P-body formation to promote viral multiplication by interaction with Ago2 and GW182. Nucleic Acids Res.

[136] Versteeg GA, Rajsbaum R, Sanchez-Aparicio MT, Maestre AM, Valdiviezo J, Shi M (2013). The E3-ligase TRIM family of proteins regulates signaling pathways triggered by innate immune pattern-recognition receptors. Immunity.

[137] Williams FP, Haubrich K, Perez-Borrajero C, Hennig J (2019). Emerging RNA-binding roles in the TRIM family of ubiquitin ligases. Biol Chem.

[138] Li YP, Duan FF, Zhao YT, Gu KL, Liao LQ, Su HB (2019). A TRIM71 binding long noncoding RNA Trincr1 represses FGF/ERK signaling in embryonic stem cells. Nat Commun.

[139] Castello A, Fischer B, Eichelbaum K, Horos R, Beckmann Benedikt M, Strein C (2012). Insights into RNA Biology from an Atlas of Mammalian mRNA-Binding Proteins. Cell.

[140] Lee H, Komano J, Saitoh Y, Yamaoka S, Kozaki T, Misawa T (2013). Zinc-finger antiviral protein mediates retinoic acid inducible gene I-like receptor-independent antiviral response to murine leukemia virus. Proc Natl Acad Sci USA.

[141] Sanchez JG, Sparrer KMJ, Chiang C, Reis RA, Chiang JJ, Zurenski MA (2018). TRIM25 Binds RNA to Modulate Cellular Anti-viral Defense. J Mol Biol.

[142] Cadena C, Ahmad S, Xavier A, Willemsen J, Park S, Park JW (2019). Ubiquitin-Dependent and -Independent Roles of E3 Ligase RIPLET in Innate Immunity. Cell.

[143] Hayman TJ, Hsu AC, Kolesnik TB, Dagley LF, Willemsen J, Tate MD (2019). RIPLET, and not TRIM25, is required for endogenous RIG-I-dependent antiviral responses. Immunology & Cell Biology.

[144] Manokaran G, Finol E, Wang C, Gunaratne J, Bahl J, Ong EZ (2015). Dengue subgenomic RNA binds TRIM25 to inhibit interferon expression for epidemiological fitness. Science.

[145] Xue B, Li H, Guo M, Wang J, Xu Y, Zou X (2018). TRIM21 Promotes Innate Immune Response to RNA Viral Infection through Lys27-Linked Polyubiquitination of MAVS. J Virol.

[146] Zhang J-F, Xiong H-L, Cao J-L, Wang S-J, Guo X-R, Lin B-Y (2018). A cell-penetrating whole molecule antibody targeting intracellular HBx suppresses hepatitis B virus via TRIM21-dependent pathway. Theranostics.

[147] Tan G, Xu F, Song H, Yuan Y, Xiao Q, Ma F (2018). Identification of TRIM14 as a Type I IFN-Stimulated Gene Controlling Hepatitis B Virus Replication by Targeting HBx. Front Immunol.

[148] Li MM, Lau Z, Cheung P, Aguilar EG, Schneider WM, Bozzacco L (2017). TRIM25 Enhances the Antiviral Action of Zinc-Finger Antiviral Protein (ZAP). PLOS Pathog.

[149] Randall G, Law LMJ, Razooky BS, Li MMH, You S, Jurado A (2019). ZAP's stress granule localization is correlated with its antiviral activity and induced by virus replication. PLOS Pathog.

[150] Sanchez-Aparicio MT, Ayllon J, Leo-Macias A, Wolff T, Garcia-Sastre A (2017). Subcellular Localizations of RIG-I, TRIM25, and MAVS Complexes. J Virol.

[151] Kumari P, Aeschimann F, Gaidatzis D, Keusch JJ, Ghosh P, Neagu A (2018). Evolutionary plasticity of the NHL domain underlies distinct solutions to RNA recognition. Nat Commun.

[152] Liu B, Li NL, Shen Y, Bao X, Fabrizio T, Elbahesh H (2016). The C-Terminal Tail of TRIM56 Dictates Antiviral Restriction of Influenza A and B Viruses by Impeding Viral RNA Synthesis. J Virol.

[153] Shen Y, Li NL, Wang J, Liu B, Lester S, Li K (2012). TRIM56 is an essential component of the TLR3 antiviral signaling pathway. J Biol Chem.

[154] Kwon SC, Yi H, Eichelbaum K, Fohr S, Fischer B, You KT (2013). The RNA-binding protein repertoire of embryonic stem cells. Nat Struct Mol Biol.

[155] Wang J, Liu B, Wang N, Lee YM, Liu C, Li K (2011). TRIM56 is a virus- and interferon-inducible E3 ubiquitin ligase that restricts pestivirus infection. J Virol.

[156] Yang P, Mathieu C, Kolaitis RM, Zhang P, Messing J, Yurtsever U (2020). G3BP1 Is a Tunable Switch that Triggers Phase Separation to Assemble Stress Granules. Cell.

[157] Sanders DW, Kedersha N, Lee DSW, Strom AR, Drake V, Riback JA (2020). Competing Protein-RNA Interaction Networks Control Multiphase Intracellular Organization. Cell.

[158] Guillen-Boixet J, Kopach A, Holehouse AS, Wittmann S, Jahnel M, Schlussler R (2020). RNA-Induced Conformational Switching and Clustering of G3BP Drive Stress Granule Assembly by Condensation. Cell.

[159] Protter DSW, Parker R (2016). Principles and Properties of Stress Granules. Trends Cell Biol.

[160] Onomoto K, Yoneyama M, Fung G, Kato H, Fujita T (2014). Antiviral innate immunity and stress granule responses. Trends Immunol.

[161] Knott GJ, Bond CS, Fox AH (2016). The DBHS proteins SFPQ, NONO and PSPC1: a multipurpose molecular scaffold. Nucleic Acids Res.

[162] Wang Y, Huang L, Wang Y, Luo W, Li F, Xiao J (2020). Single-cell RNA-sequencing analysis identifies host long noncoding RNA MAMDC2-AS1 as a co-factor for HSV-1 nuclear transport. Int J Biol Sci.

[163] Wyler E, Menegatti J, Franke V, Kocks C, Boltengagen A, Hennig T (2017). Widespread activation of antisense transcription of the host genome during herpes simplex virus 1 infection. Genome Biology.

[164] Rutkowski AJ, Erhard F, L'Hernault A, Bonfert T, Schilhabel M, Crump C (2015). Widespread disruption of host transcription termination in HSV-1 infection. Nat Commun.

[165] Guo CJ, Ma XK, Xing YH, Zheng CC, Xu YF, Shan L (2020). Distinct Processing of lncRNAs Contributes to Non-conserved Functions in Stem Cells. Cell.

[166] Hosono Y, Niknafs YS, Prensner JR, Iyer MK, Dhanasekaran SM, Mehra R (2017). Oncogenic Role of THOR, a Conserved Cancer/Testis Long Non-coding RNA. Cell.

[167] Fiorenzano A, Pascale E, Gagliardi M, Terreri S, Papa M, Andolfi G (2018). An Ultraconserved Element Containing lncRNA Preserves Transcriptional Dynamics and Maintains ESC Self-Renewal. Stem Cell Rep.

[168] Lin N, Chang KY, Li Z, Gates K, Rana ZA, Dang J (2014). An evolutionarily conserved long noncoding RNA TUNA controls pluripotency and neural lineage commitment. Mol Cell.

[169] Sun Y, Cai M, Zhong J, Yang L, Xiao J, Jin F (2019). The long noncoding RNA lnc-ob1 facilitates bone formation by upregulating Osterix in osteoblasts. Nat Metab.

[170] Bolha L, Ravnik-Glavac M, Glavac D (2017). Long Noncoding RNAs as Biomarkers in Cancer. Dis Markers.

[171] Meng L, Ward AJ, Chun S, Bennett CF, Beaudet AL, Rigo F (2015). Towards a therapy for Angelman syndrome by targeting a long non-coding RNA. Nature.

[172] Kulkarni S, Lied A, Kulkarni V, Rucevic M, Martin MP, Walker-Sperling V (2019). CCR5AS lncRNA variation differentially regulates CCR5, influencing HIV disease outcome. Nat Immunol.

[173] Balwani M, Sardh E, Ventura P, Peiro PA, Rees DC, Stolzel U (2020). Phase 3 Trial of RNAi Therapeutic Givosiran for Acute Intermittent Porphyria. N Engl J Med.

[174] Xiao R, Chen JY, Liang Z, Luo D, Chen G, Lu ZJ (2019). Pervasive Chromatin-RNA Binding Protein Interactions Enable RNA-Based Regulation of Transcription. Cell.

